# Immediate and long-term transcriptional response of hind muscle tissue to transient variation of incubation temperature in broilers

**DOI:** 10.1186/s12864-016-2671-9

**Published:** 2016-05-04

**Authors:** Watcharapong Naraballobh, Nares Trakooljul, Eduard Muráni, Ronald Brunner, Carsten Krischek, Sabine Janisch, Michael Wicke, Siriluck Ponsuksili, Klaus Wimmers

**Affiliations:** Leibniz Institute for Farm Animal Biology (FBN), Institute for Genome Biology, 18196 Dummerstorf, Germany; Institute of Food Quality and Food Safety, University of Veterinary Medicine Hannover, D-30173 Hannover, Germany; Department of Animal Science, Quality of Food of Animal Origin, Georg-August-University Goettingen, D-37075 Goettingen, Germany

**Keywords:** Gene expression, Pathway analysis, *In-ovo* development, Poultry, Microarray

## Abstract

**Background:**

In oviparous species accidental variation of incubation temperatures may occur under natural conditions and mechanisms may have evolved by natural selection that facilitate coping with these stressors. However, under controlled artificial incubation modification of egg incubation temperature has been shown to have a wide-ranging impact on post-hatch development in several poultry species. Because developmental changes initiated *in-ovo* can affect poultry production, understanding the molecular routes and epigenetic alterations induced by incubation temperature differences may allow targeted modification of phenotypes.

**Results:**

In order to identify molecular pathways responsive to variable incubation temperature, broiler eggs were incubated at a lower or higher temperature (36.8 °C, 38.8 °C) relative to control (37.8 °C) over two developmental intervals, embryonic days (E) 7–10 and 10–13. Global gene expression of *M. gastrocnemius* was assayed at E10, E13, and slaughter age [post-hatch day (D) 35] (6 groups; 3 time points; 8 animals each) by microarray analysis and treated samples were compared to controls within each time point. Transcript abundance differed for between 113 and 738 genes, depending on treatment group, compared to the respective control. In particular, higher incubation temperature during E7-10 immediately affected pathways involved in energy and lipid metabolism, cell signaling, and muscle development more so than did other conditions. But lower incubation temperature during E10-13 affected pathways related to cellular function and growth, and development of organ, tissue, and muscle as well as nutrient metabolism pathways at D35.

**Conclusion:**

Shifts in incubation temperature provoke specific immediate and long-term transcriptional responses. Further, the transcriptional response to lower incubation temperature, which did not affect the phenotypes, mediates compensatory effects reflecting adaptability. In contrast, higher incubation temperature triggers gene expression and has long-term effects on the phenotype, reflecting considerable phenotypic plasticity.

**Electronic supplementary material:**

The online version of this article (doi:10.1186/s12864-016-2671-9) contains supplementary material, which is available to authorized users.

## Background

Chickens and other birds are homeotherms that require that their body temperatures are maintained within a limited range during pre- and post-hatch processes [1]. Altering the temperature range during the critical developmental periods may cause only minor morphological differences, or could even produce lethal events. Since under natural conditions unpredictable periods may occur when incubation temperatures are unfavorable, natural selection could have promoted traits and mechanisms that provide resilience against such exogenous factors and that are reflected by immediate, acute or long-term, delayed responses (Du and Shine, 2015). Shifts in the incubation temperature of eggs under controlled experimental conditions have been shown to impact post-hatch development in several bird species. However, results of previous studies are inconsistent. For example, a higher incubation temperature was concluded to positively affect breast meat yield in featherless broilers [2] and muscle fiber size in turkey [3], but was associated with body weight loss in live chicken [4]. Similarly, lower incubation temperature was indicated to have a prolonged effect on female embryo mortality in Australian Brush-turkey [5], but reportedly reduced growth rates of wood duck [6]. Thus, the effects of incubation temperature changes on post-hatch development remain unclear. In particular, there is a lack of studies addressing the response to exogenous physical effects on the level of gene expression that will promote the understanding of the underlying compensatory, adaptive and regulatory process that might be associated with the treatment.

**Fig. 1:**
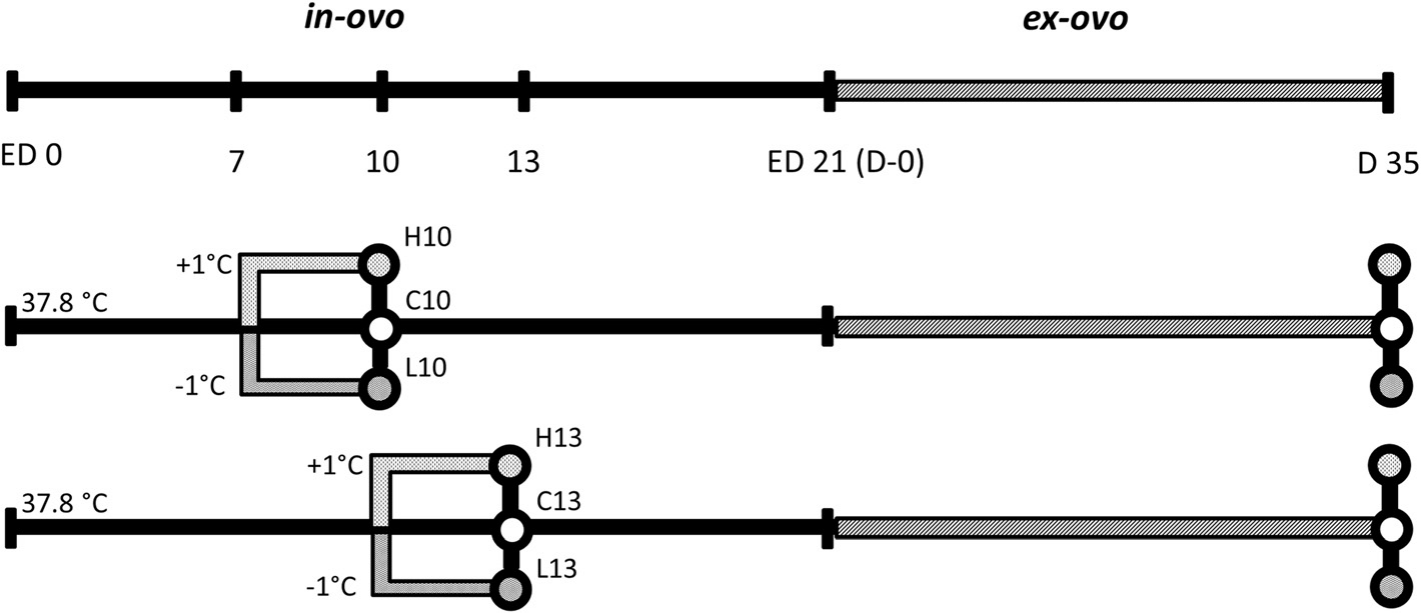
Experimental design; indicating the two periods of modulated incubation temperatures at E7-10 and E10-13 and the time points of samplings (88 samples; *n* = 8 per treatment with D35-controls *n* = 8 in total)

The *in-ovo* development of birds offers a valuable model in which to study environmental effects on myogenesis. Indeed, the identification of shifts in muscle and growth traits facilitates the detection of candidate genes for these traits. During avian myogenesis, the muscle fibers are formed in two phases. The primary muscle fiber, which is a core fiber, transforms to a myotube between the 4^th^ and 7^th^ embryonic days (E). Next, secondary muscle fibers, which are smaller and derive from myoblasts, arrange around the primary muscle fiber as a scaffold, proceeding until E15 [7]. Fetal myoblasts are most abundant between E8 and E12 [8]. After the secondary phase, depending on morphology and localization of the myofibers, the adult myoblasts will transform and become the primary source of myogenic precursors for postnatal muscle formation [9–11]. During both critical stages, temperature manipulation may cause differential expression of genes to produce phenotypic changes. Previous studies showed that elevated incubation temperature over E7-10 positively influenced carcass traits in broiler males, but did not affect meat quality [12]. Thus, shifting the incubation temperature during targeted periods of *in-ovo* development could contribute to the improvement of the efficiency of broiler meat production, without sacrificing meat quality. This study addresses the transcriptomic response of skeletal muscle tissue to transient reduction and elevation of incubation temperature at early (E7-10) and late (E10-13) secondary muscle fiber development. Microarray expression profiles of treated samples were compared to those of the respective controls immediate after the treatment periods (E7-10; E10-13) and also later at slaughter (Fig. 1). The results have implication for the molecular foundation of potential impact on meat production traits and also provide insight into the mechanisms involved in the resilience against low and the phenotypic plasticity against high incubation temperature.

## Results

### Global gene expression pattern of chicken hind muscle

The chicken gene 1.0 ST array contains 165,815 probe-sets representing 20,828 transcripts encoding for 18,214 genes. After quality filtering and normalization, probe-sets representing 8,909 transcripts were subjected to further analyses. Analysis of variance was used to identify differentially expressed genes (DEGs) by comparing gene expression levels of treatment group (*in-ovo* temperature modification) against the control. The number of DEGs for each comparison at embryonic stages and at D35 is shown in Table 1. At the embryonic stage, higher temperature during E7-10 versus control (H10ΔC, 38.8 °C) significantly altered the expression of 738 genes compared to other treatment conditions (Table 1). Lower temperature during E10-13 versus control (L13ΔC, 36.8 °C) affected more genes than did low temperature during E7-10 (389 vs 140). Long-term effects of the *in-ovo* temperature modification were investigated at D35. Lower temperature in the early and late treatment period (L10ΔC and L13ΔC) resulted in a high number of DEGs at D35 (693 and 288, respectively), whereas higher temperature produced fewer DEGs at D35 (167 and 247, respectively) (Table 1). In addition, the majority of DEGs were down-regulated in embryonic stage, but were up-regulated at D35, as shown in Table 1. The direction of regulation of FGA, NR4A3 and AHSG, exemplarily chosen as to represent genes assignment to several pathways, as indicated by microarrays and qPCR were consistent. The correlation coefficients were highly significant and ranged between 0.71 and 0.84. Taken together, the qPCR analyses indicated a reproducible analysis.

**Table 1:** Numbers of differentially expressed probes sets and respective genes (DEGs); comparisons between each *in-ovo* thermal modification condition the time-matched control separated for embryonic stages or at D35 (*p* ≤ 0.05)

	Treatment (ΔC)	Probe sets	DEGs	Regulation	
				Up	Down
Embryo	H10 - C10	812	738	662	76
	H13 - C13	176	113	88	25
	L10 - C10	169	140	34	106
	L13 - C13	503	389	258	131
D35	H10 - C10	217	167	35	132
	H13 - C13	332	247	108	139
	L10 - C10	768	693	104	589
	L13 - C13	330	288	123	165

### Distinct response to temperature alteration by time and direction

**Fig. 2:**
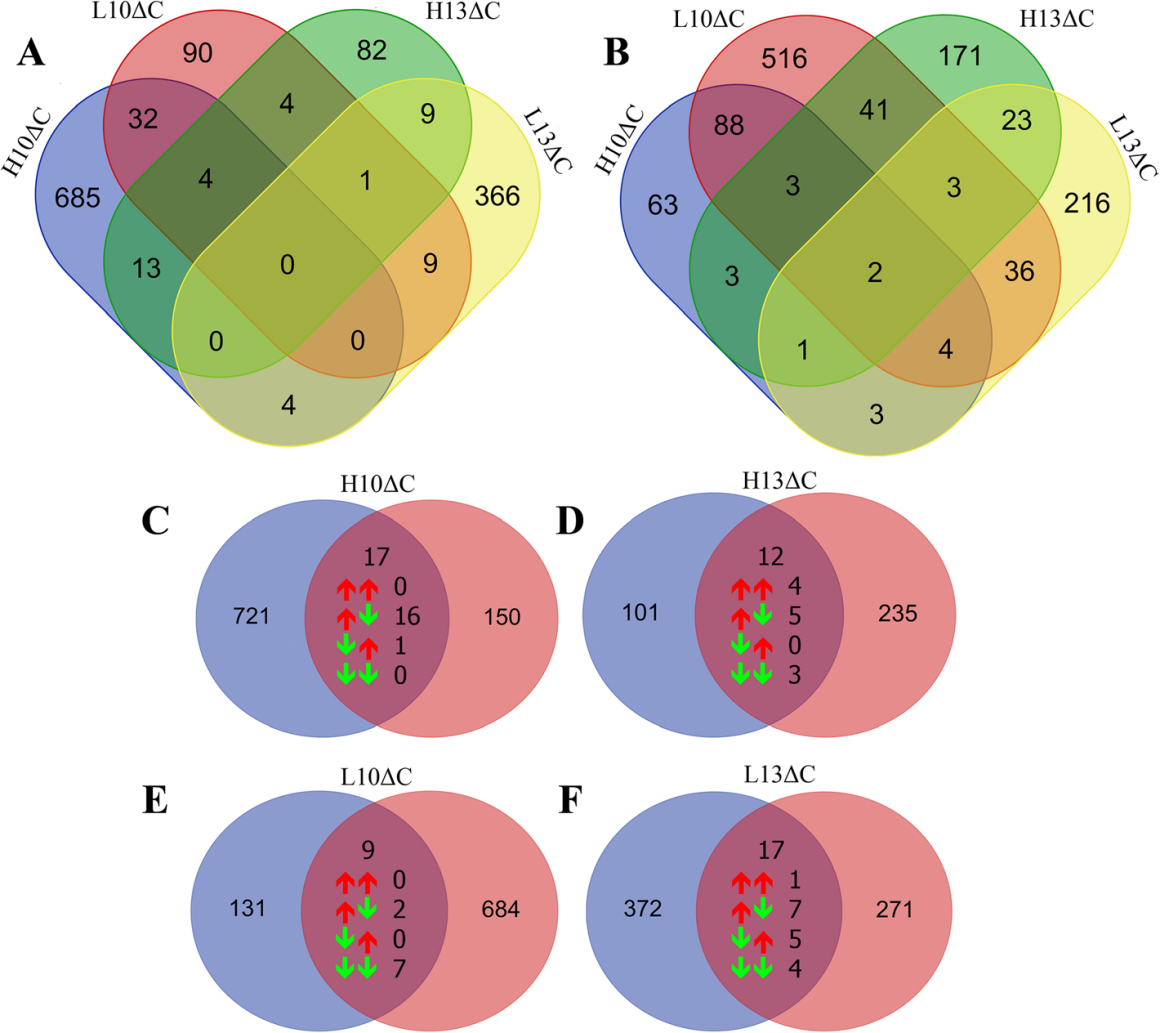
Venn diagrams displaying numbers of differentially expressed genes for each treatment condition relative to control. Comparisons between treatment conditions at embryonic stages (**a**) and at D35 (**b**) as well as between embryonic stages and D35 after the same treatments (**c**) (blue embryonic, red D35)

A comparison of the DEGs between treatment conditions showed that most DEGs were unique for each condition, e.g., 685 and 366 DEGs for H10ΔC and L13ΔC in the embryonic stages (Fig. 2a), and 516 and 216 DEGs for L10ΔC and L13ΔC at D35 (Fig. 2b), respectively. Some DEGs were shared between two conditions, including those common either to L10 and L13 (10 and 45 at embryonic stage and D35) or to H10 and H13 (17 and 9 at embryonic stage and D35), which were almost exclusively consistently regulated. There were only a few DEGs that were common across more than 2 conditions. Comparisons of DEGs from identical treatments in embryonic and D35 samples (Fig. 2c) revealed 55 common DEGs in total for the 4 treatments. Of these, 5 up- and 14 down-regulated transcript ids were regulated in the same direction in both embryonic stages and at D35. However, most of the common DEGs were regulated in opposite directions by stage, e.g., up-regulation in embryos and down-regulation at D35. A list of common DEGs with fold-change and *p*-value is available in Additional file 1.

### Pathway analysis

**Fig. 3:**
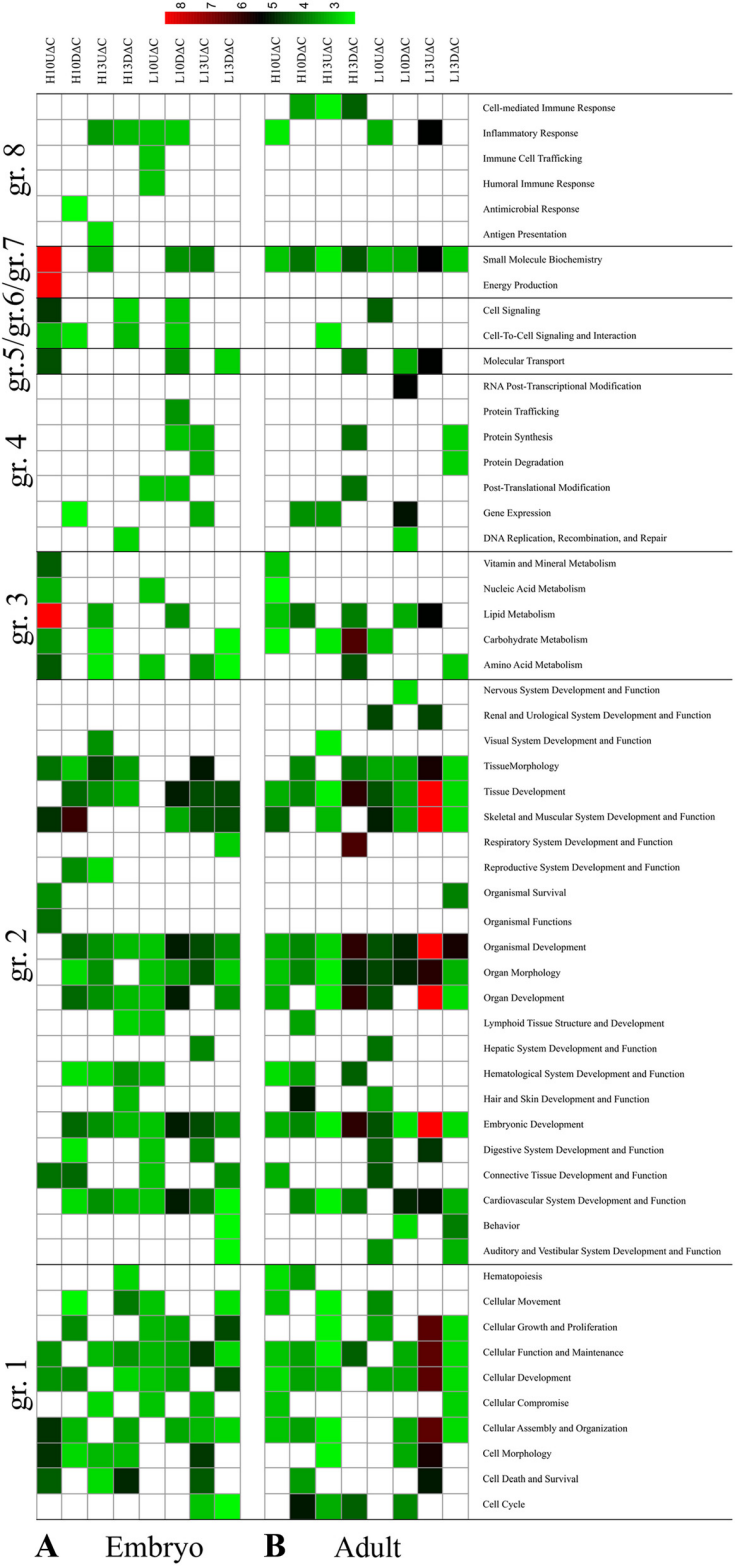
Significant pathways altered by *in-ovo* thermal modifications; in (**a**) embryonic stage and (**b**) D35. DEGs associated with each comparison (treatment vs control) are separated into up-regulation (U) or down-regulation (D). Thermal modification treatments: increase (H) or decrease (L) incubation temperature during E7-10 (H10 and L10) or E10-13 (H13 and L13). Significant pathways (IPA defined) are grouped into eight major categories of interest; group (gr.)1 cell maintenance proliferation differentiation and replacement, gr.2 organismal organ and tissue development, gr.3 nutrient metabolism, gr.4 genetic information and nucleic acid processing, gr.5 molecular transport, gr.6 cell signaling and interaction, gr.7 small molecule biochemistry, and gr.8 response to stimuli and associated. The –log (BH *p*-value) associated with significant pathways are plotted in green (small) to red (large)

To understand the underlying biology and identify relevant pathways, DEGs were analyzed using the Ingenuity Pathway Analysis software tools (IPA, Ingenuity Systems Inc., Redwood City, USA). All DEGs lists were separated into up- or down-regulated genes for each comparison (temperature modification vs control). All significant biological pathways associated with *p*-values and gene members are available in Additional files 2, 3, 4 and 5. The pathway analysis approach is effective for handling a list of DEGs, and generates a list of biological terms/pathways. To encompass most pathways affected by all treatment factors (temperature modification, embryonic stage, and growth stage), we grouped 52 and 49 significant pathways derived from embryos and D35 broiler, respectively, into eight major categories of interest: group (gr.) 1, cell maintenance, proliferation, differentiation and replacement; gr.2, organismal, organ, and tissue development; gr.3, nutrient metabolism; gr.4, genetic information and nucleic acid processing; gr.5, molecular transport; gr.6, cell signaling and interaction; gr.7, small molecule biochemistry; and gr.8, response to stimuli. Overall, biological pathways involved in cell growth (gr.1) and tissue development (gr.2) were affected by modification of incubation temperature both in embryos and at D35 based on the number of pathways, as shown in Fig. 3. Activation states of upstream regulators were further analyzed for the dataset based on the Z-score calculation from Ingenuity Pathway Analysis (IPA) (Additional files 2-5).

In particular, a higher temperature during E7-10 (H10ΔC; Fig. 3a, rows 1&2) altered pathways involved in lipid metabolism, cell signaling, energy metabolism, muscle development and function, and small molecule biochemistry, more so than did other conditions in embryos (Fig. 3a, rows 3–8). Z-scores indicate that H10ΔC condition tended to activate several pathways related to nutrient metabolism (gr.3) and small molecule biochemistry (gr.7) (Additional file 2). A lower temperature (L10ΔC and L13ΔC; Fig. 3a, rows 5–8) affected pathways related cell maintenance, proliferation, differentiation and replacement (gr.1) and organismal, organ, and tissue development (gr.2). L13ΔC tended to suppress cellar processes related to cell death, thus promoting maintenance in the major category cell maintenance, proliferation, differentiation and replacement (gr.1) and to activate developmental processes in mesoderm and muscle (gr.2, organismal, organ, and tissue development) (Additional file 3).

**Fig. 4:**
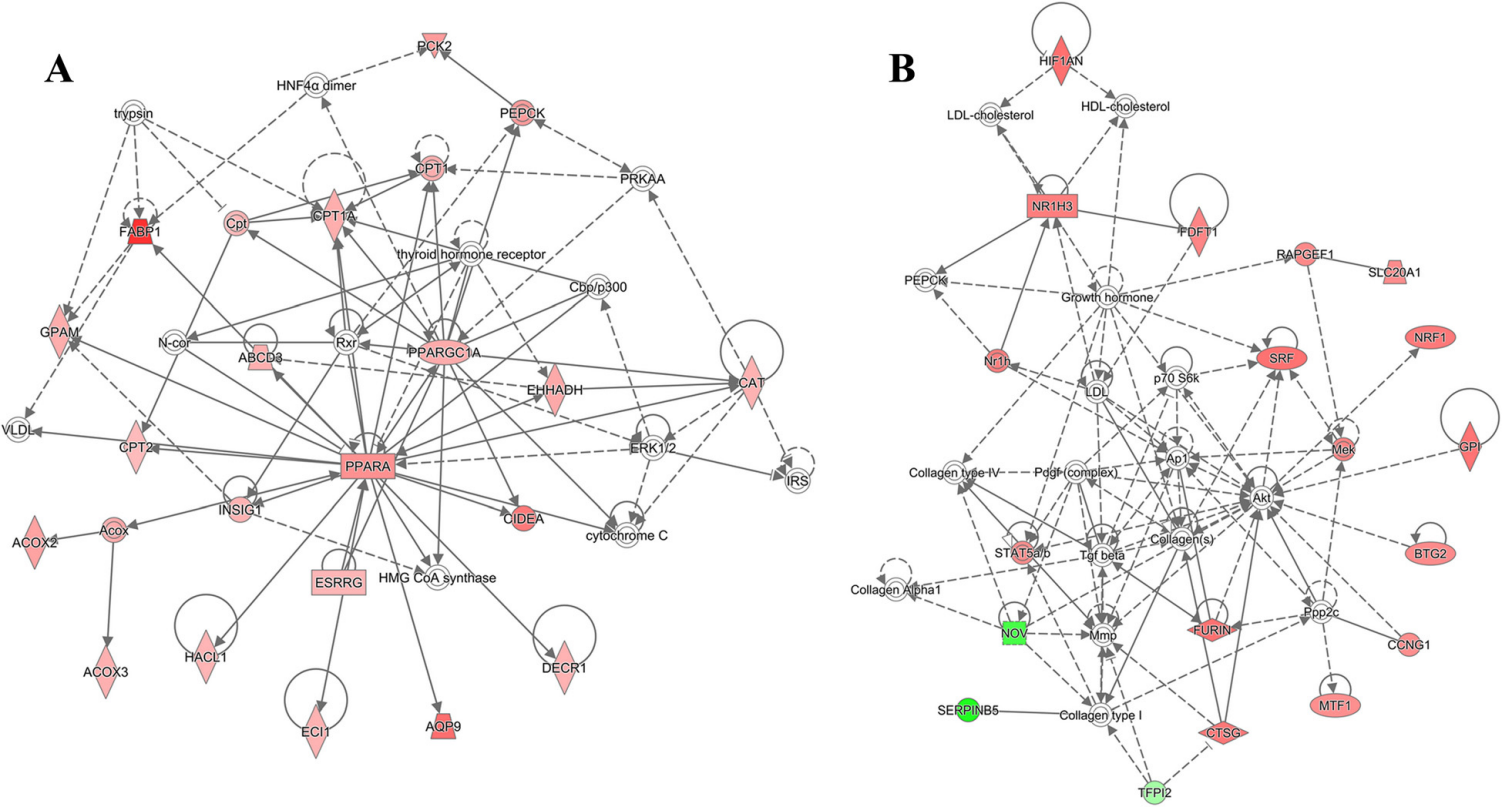
Functional networks derived from sets of DEG obtained for H10ΔC (**a**) and L13ΔC (**b**) at embryonic stage. Based on the Ingenuity KnowledgeBase a network of up regulated genes was derived for H10ΔC indicating activated energy production, lipid metabolism and small molecule biochemistry. For L13ΔC a network was found implying deactivated cell death and survival, but activated cellular growth and proliferation pathways. Red and green indicate up- and down-regulation; network shapes indicate various classes of network components; line and arrows indicate undirected and directed interactions

Using all DEGs obtained for H10ΔC in embryos, a network was generated covering 19 DEGs. *FABP1* (fatty acid binding protein 1), *PPARA* (peroxisome proliferator-activated receptor alpha), and *PPARGC1A* (peroxisome proliferator-activated receptor gamma, coactivator 1 alpha) are highly connected genes in the network and related to energy production, lipid metabolism and small molecule biochemistry (Fig. 4a). For L13ΔC, the generated network was related to suppressed cell death and survival but stimulated cell growth and digestive developmental processes, including genes *GPI* (glucose-6-phosphate isomerase), *NR1H3* (nuclear receptor subfamily 1, group H, member 3), and *SRF* (serum response factor) (Fig. 4b).

At D35, decreased incubation temperature during E10-13 (L13ΔC; Fig. 3b, rows 7&8) strongly changed pathways related to cellular function and growth development of organs, tissue and muscle as well as nutrient metabolism pathways (Fig. 3b). For L10ΔC (Fig. 3b, rows 5&6) a considerable number of genes were affected that belong to Ingenuity biological functions related to organismal, organ, and tissue development (gr.2). Notably, according to Z-scores L10ΔC tended to exhibit inhibitory effect on genetic processing categories (gr.4; Genetic information and nucleic acids), whereas L13ΔC was more likely to activate most categories (Additional file 4). For H13ΔC (Fig. 3b, rows 3&4) broadly the same molecular routes were shifted, however in opposite direction (Fig. 3b). For H10ΔC, no trends of activation or inhibition of pathways were obvious (Additional file 5).

**Fig. 5:**
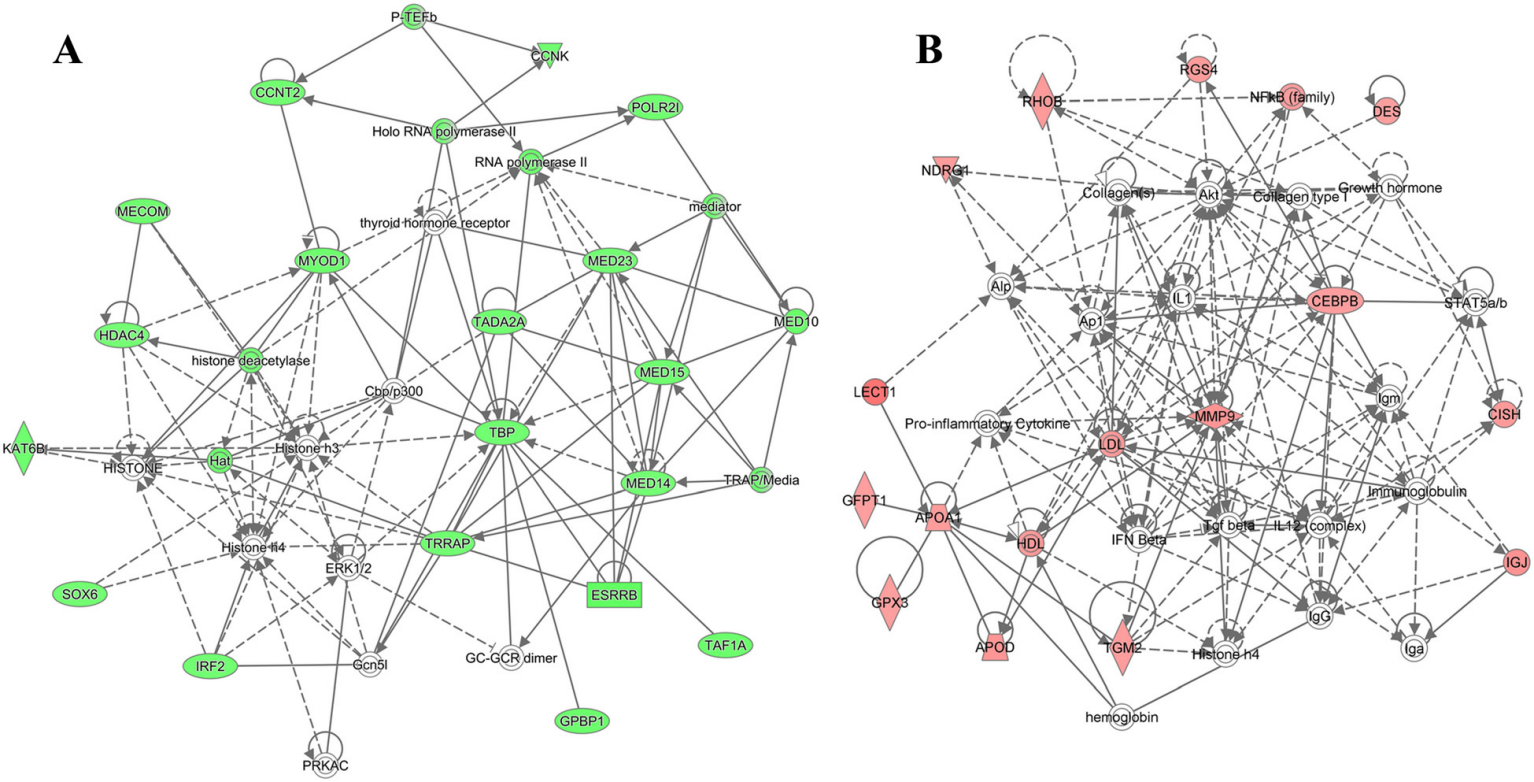
Functional networks derived from sets of DEG obtained for L10ΔC (**a**) L13ΔC (**b**) at D35. Based on the Ingenuity KnowledgeBase a network of down regulated genes was derived for L10ΔC indicating deactivated gene expression, cellular function and organismal development networks. For L13ΔC a network was found implying deactivated tissue development, skeletal and muscular disorders and cell-to-cell signaling pathways. Red and green indicate up- and down-regulation; network shapes indicate various classes of network components; line and arrows indicate undirected and directed interactions

The network established for L10ΔC contained genes of top pathways including gene expression, cellular function and maintenance, and organismal development (Fig. 5a). Highly connected genes included *HDAC4* (histone deacetylase 4), *TBP* (TATA Box Binding Protein), *MYOD1* (myogenic differentiation 1), and *SOX6* (sex determining region Y-box 6) that are related to inactivation of transcription and muscle cell differentiation. For L13ΔC, activation of pathways related to proliferation, differentiation, and development at the cell, tissue, and organ levels was predicted. Accordingly, the consistently increased transcript abundances revealed a network (Fig. 5b). The involved genes for nutrition metabolism included *APOA1* (apolipoprotein A1), *GFPT1* (glutamine fructose-6-phosphate transaminase 1) and proliferation of muscle development included *APOD* (apolipoprotein D), and *DES* (desmin).

## Discussion

This study demonstrates that transcriptomic and pathway regulation changes occur in broiler embryos and at D35 as a result of temperature manipulation during early (E7-10) and later (E10-13) development stage. Evidence was reported that early elevated incubation temperature positively influenced growth traits, but did not affect meat quality [12]. Indeed the chicken analyzed here showed slight but significant increase in body weight when transiently incubated at higher temperature, whereas decreased incubation temperature did not affect body weight.

### Immediate effects observed *in-ovo*

Embryonic days 7–10 and 10–13 cover the final stage of primary muscle formation and start of secondary muscle fiber formation, respectively [7]. During myogenesis, multiple transcripts have major roles in regulating muscle development, such as *BCK* (B isoform of creatine kinase) [13], *cTnT* (chicken cardiac troponin T) [14], *Mstn* (myostatin), and *MyoD* (myogenic differentiation 1) [15]. Previous research showed many regulated genes expressed during myogenesis being sensitive to incubation temperature manipulation.

### Immediate response to early high temperature treatment (H10ΔC)

Due to our experiment, the major impacts on the transcriptome resulted from early high (H10ΔC) and late low (L13ΔC) temperature shifts, with the majority of DEGs being up regulated. The H10ΔC comparison indicated that pathways involved in lipid metabolism, energy production, oxidation and beta-oxidation of fatty acid were activated. In this network, 19 up-regulated DEGs were represented, including *FABP1, PPARA,* and *PPARGC1A*.

*FABP1* and PPARA act in fatty acid uptake, metabolism, and intracellular transportation of lipids metabolism, cell proliferation, cell differentiation and respiration as well as inflammation responses [16]. A previous study showed that *L-FABP* in chicken had higher expression in fat-type chicken at 3, 5, and 7 weeks old (*p*-value ≤ 0.05), and is linked to abdominal fat deposition and high lipogenesis rate [16]. Moreover, a previous study showed shifts of expression of *AMPK-PPARA* pathway genes due to thermal conductions [17]. *PPARGC1*, regulates energy metabolism, muscle fiber specialization and adaptive thermogenesis [18–20]. A previous study reported that single nucleotide polymorphisms (SNPs) in chicken *PPARGC1A* are significantly related to abdominal fat weight without growth trait effects [21]. Moreover, *PPARGC1A* (*PGC-1α*) which was influenced by cold-stress (4 °C from D28 to D38) in chicken could influenced a change in fiber type distribution and phenotype [20]. Exemplarily, modulated expressions of these genes reflect shifts of biological functions related to growth and metabolism.

The results demonstrate immediate shifts of transcript abundance due to manipulation of incubation temperature. During E7-10, high temperature (38.8 °C) manipulation influenced mainly lipid (*FABP1, PPARA*) and energy production (*PPARGC1*) pathway. Accordingly, changes of body, liver, and heart weight were evident [22]. Moreover, activity of mitochondrial respiration (state-3-pyruvate/malate and state-3-succinate/rotenone) and enzyme activities (glycogen phosphorylase, lactate dehydrogenase, and cytochrome oxidase) were elevated [22]. Thus, H10 treatment could influence lipid production and metabolism and also promoted phenotypic change.

### Immediate response to late low temperature treatment (L13ΔC)

At later development (E10-13), low temperature had a greater impact on the transcriptome (L13ΔC). The biological functions affected were predicted to affect cellular processes balancing differentiation, proliferation and maintenance. In contrast, high temperature treatment down regulated the development of cytoplasm and vasculogenesis, but increased inflammation and cell death. The L13ΔC network related to cell death and survival and cellular growth and proliferation. Candidate DEGs included *GPI, NR1H3,* and *SRF,* involved in metabolic, proliferation, and differentiation pathways.

In fact *GPI* encodes a member of the glucose phosphate isomerase protein family, involved in glucose metabolism [23]. In chicken, GPI is up-regulated in muscle development [24]. *NR1H3* belongs to the *NR1* subfamily of the nuclear receptor superfamily (synonym: liver X receptor alpha), which are key regulators of macrophage function, inflammation, and lipid homeostasis in differentiating chondrocytes [25, 26]. In chicken, *NR1H3* is considered a key regulator of fatty acid homeostasis [27] and cholesterol homeostasis [25]. *SRF* encodes a ubiquitous nuclear protein that stimulates cell proliferation and differentiation. In chicken embryo, *SRF* expression is restricted primarily to striated muscle cell lineages, which increased mass of nuclear and activating alpha-actin gene activity [28].

Later in E10-13, lower temperature (36.8 °C) was associated with shifts of pathways towards balancing anabolic and catabolic pathways, which is in line with phenotypic change being slight and non-significant [22]. Enzyme activity (cytochrome oxidase) and mitochondrial respiration (state-3-pyruvate/malate) were lower than at normal condition. It was suggested that lower temperature at late treatment might decelerate embryonic activity.

### Long-term effects observed at D35

In-ovo shifts of thermal conditions had long term effects on the transcriptome observed at D35. Higher or lower incubation temperature has also been shown to impact postnatal development in avian species [3, 6, 29]. Because the egg shell temperature is sensitive to environmental change, it can directly impact developmental processes as well [30, 31]. Acute temperature modulation at the late embryonic stage was previously suggested to cause long-term transcriptomic changes, but few studies demonstrated an ongoing effect. A recent report showed embryonic temperature manipulation affected thermoregulatory mechanisms [32]. Another study found that periodic incubation temperature change between 37.8 °C and 39.5 °C from E16-18 initiated acute (E17) and late-term (D13 post-hatch) positive effects on diameter of myofibers and muscle cell proliferation in chicken [1]. These findings might have been resulted from modifications to the stress response and thermogenesis by the increased temperature from E7-16, resulting in reduced oxygen consumption, heart rate, and egg shell temperature. These changes directly affected broiler embryo growth and development [33]. Similarly, another study showed that temperature manipulation caused a high density of blood vessels in the chorioallantoic membrane during embryogenesis [34]. In our studies, long-term transcriptomic changes were due to low temperature treatments (L10ΔC and L13ΔC conditions) primarily leading to down-regulation. IPA analysis indicated that increasing the incubation temperature to 38.7° may influence cell cycle and skin development at the early time point (E7-10). After that, high temperature E10-13 treatment tended to activate apoptosis in cell development but deactivated cardiovascular system and body trunk. Effects on metabolic process showed a reduction of carbohydrate metabolism, synthesis. Furthermore, a negative effect still remained for concentration of lipid and acylglycerol.

### Long-term response to early low temperature treatment (L10ΔC)

The lower incubation temperature resulted in more DEGs in both early and late treatments. Early low temperature (L10ΔC) tended to activate pathways involved in organismal development and cell proliferation but strongly suppress transcriptional process. All significant pathways including gene expression, cellular function and maintenance, and organismal development formed a network. Candidate genes included *HDAC4, MYOD1,* and *SOX6,* which are related to inactivation of transcription and muscle cell differentiation.

Previous research showed *HDAC4* was associated in modulating cell growth and differentiation by controlling histone deacetylase activity, which alters chromosome structure and affects transcription factor access to DNA [35, 36]. A negative effect of *HDAC4* overexpression is down-regulation of cardiac muscle gene expression and leads to inhibition of cardiomyogenesis [37]. Normally *HDAC4* was found in neuromuscular junction especially in myonuclei of fast oxidative skeletal muscle fibers [38, 39]. Down regulated of this gene suggested multiple transcriptional abnormalities including cardiac hypertrophy [40] and influence to *MYOD1* expression. *MYOD1* encodes a protein that belongs to a basic helix-loop-helix family of transcription factors and the myogenic factors subfamily. Generally, *MYOD1* acts in muscle cell differentiation by inducing cell cycle arrest. During pre-gastrulating epiblast in chicken, *MYOD1* can induce skeletal muscle lineage self-renewal and differentiation [41]. Moreover, *MYOD1* also works with the downstream effector *VGL-2* in skeletal myogenesis [42]. Another DEGs which suggested to downregulate in cardiac and skeletal muscle is *SOX6*, a member of the *SOXD* gene family, encodes functional domains including a DNA binding domain (the HMG box) and two coiled-coil domains [43]. The encoded protein is a transcriptional activator and critical role in cartilage development and mesenchymal differentiation [44]. Moreover, *SOX6* is well known to function as a transcriptional suppressor of slow fiber-specific genes [45, 46].

Lowered incubation temperature had large effects on postnatal expression in terms of number of transcripts with shifted abundance. Manipulation of early treatment (E7-10) led to down regulation in transcriptional processes and muscle cell differentiation. Moreover, cardio (*HDAC4*) and skeletal (*MYOD1 and SOX6*) myogenesis were negatively affected. The phenotype of D35 chicken exposed to lower temperature showed a slight non-significant reduction of carcass and leg compared to the control group; higher incubation temperature led to increased weights (Additional file 6).

### Long-term response to late low temperature treatment (L13ΔC)

Late low temperature treatment (L13ΔC) was predicted to activate pathways in cellular and organismal development including cell survival, development of body trunk, contractility of cardiac muscle, and proliferation of mammary epithelial cells, but to have a negative effect on size of body and muscle cell pathways. In metabolism, elevated uptake and metabolism of lipid and carbohydrate, together with small molecule biochemistry like oxidation of fatty acid, tended to reduce concentration of lipid. Inflammatory response was also predicted to be suppressed. The IPA network highlighted the activation of tissue development, skeletal and muscular disorders, and cell-to-cell signaling. Selection of Fold change (FC) related in every major category revealed a set of candidate genes: *APOD*, *APOA1*, *DES,* and *GFPT1*.

*APOD* encodes a component of high-density lipoprotein (HDL) with a high degree of homology to plasma retinol-binding protein and lipocalins. During late chicken embryogenesis, the expression of *APOD* is enriched among subsets of central nervous system (CNS) neurons then again in skin during developing of feather [47]. The molecular function involved lipoprotein metabolism, as shown by *APOA1*, *HDL,* and *LDL* in the network Fig. 5b. *APOA1* is the major protein component of high-density lipoprotein in plasma. It promotes cholesterol efflux from tissues to the liver for excretion, and is a cofactor for lecithin cholesterol acyl transferase (*LCAT*), which is responsible for the formation of most plasma cholesteryl esters. *APOA1* is negatively correlated with aging and influences muscle development in Thai indigenous chicken [48]. Desmin (*DES*) encodes a muscle-specific class III intermediate filament. Homopolymers of this protein form a stable intra-cytoplasmic filamentous network connecting myofibrils to each other and to the plasma membrane. It maintains the structural integrity of highly solicited skeletal muscle and is important to other biological processes including muscle contraction and development, especially in heart contraction [49–51]. *GFPT1* controls the flux of glucose into the hexosamine biosynthetic pathway, providing building blocks for the glycosylation of proteins and lipids [52]. The product of this gene catalyzes the formation of glucosamine 6-phosphate, which participates in carbohydrate biosynthesis and apoptosis regulation. *GFPT1* is expressed in many tissues including skeletal muscle and heart [52]. Network connection revealed discreet interaction between *GFPT1* and *APOA1*.

Low incubation temperature at late treatment (L13) had an impact on multiple DEGs and pathways in both embryo stages and at D35. However, these transcriptomic changes were not associated with significant phenotypic changes compared to the control (Additional file 6). The transcriptional response to lower incubation temperature appears to mediate compensatory effects that indicate a considerable adaptability. In nature transient reduction of incubation temperature during natural brooding happens. Accordingly, regulatory mechanism evolved in birds that enable the emergence of normal phenotypes. In contrast, higher incubation temperature triggers gene expression and has long-term effects on the phenotype. Elevated temperature is not likely in natural brooding, consequently not compensatory mechanisms evolved. Phenotypic changes associated with increased incubation temperature display metabolic plasticity of chicken.

## Conclusions

Our experiment shows that manipulation of incubation temperature immediately effected transcriptomic changes and influenced the long-term expression. In parallel the results on growth, carcass, meat quality and mitochondrial respiratory activities indicate effects of transient variation of incubation temperature as well [22, 53]. The observations indicate the successful activation of compensatory mechanisms in adaptation to lowered temperature and phenotypic plasticity in response to elevated temperature. Further investigations of the mechanism behind these regulatory processes including epigenetic modifications provide the perspective to improve resistance to environmental changes without much effect on growth performance [32]. Moreover, numerous genes which play important roles in metabolic pathways and which showed changed expression due to shifted incubation temperature represent candidate genes for further genetic improvement in terms of resilience against temperature shifts or in terms of increased muscle growth without affecting meat quality.

## Methods

### Animals and tissue collection

As outlined in Fig. 1, hatching eggs of a commercial broiler line (Cobb-Vantress Inc., Siloam Springs, USA) were randomly assigned to the following experimental groups: H10 and L10, which were subjected to higher (38.8 °C) or lower (36.8 °C) incubation temperature, respectively, between E7-10; and H13 and L13, which were subjected to the same temperature shifts, respectively, but between E10-13. During the rest of the incubation period, all eggs were incubated at 37.8 °C, like the control group (C10, C13). Samples were collected immediately at the end of the treatment periods at E10 and E13, respectively, and in addition at post-hatch at day 35. The hatchlings were reared in barn system and fed a standard diet *ad libitum* until day 35 (D35; slaughter). Samples of hind tissues (*M. gastrocnemius*) were collected and immediately stored in liquid nitrogen. Embryonic samples taken at ED10 and ED13 as well as samples of D35 were sexed and for each experimental group (C10, H10, L10 and C13, H13, L13) at each time point (E10 or E13, respectively, plus D35) samples, balanced for sex, were selected for gene expression analyses with 8 samples per treatment (Fig. 1). The recording of zoo-technical and biochemical traits was performed at the end of the respective treatment periods [53]. Increased incubation temperature led to slight but significant differences in body weight and mitochondrial respiratory capacity, whereas decreased incubation temperature only had subtle effects on a few parameters (Additional file 6). The study was approved by the institutional Animal Welfare Committees and was conducted according to the guidelines of the German Law of Animal Protection.

### RNA isolation

Total RNA of frozen individual tissue samples was isolated with Tri-Reagent-extraction (Sigma-Aldrich, Taufkirchen, Germany) according to manufacturer’s protocol. DNase treatment and a column-based purification using the RNeasy Mini Kit (Qiagen, Hilden, Germany) were also performed according to manufacturers’ protocols. To check RNA integrity, samples were visualized on 1 % agarose gels containing ethidium bromide. RNA concentration was determined by spectrometry with a NanoDrop ND-1000 spectrophotometer (PEQLAB, Erlangen, Germany). The absence of DNA contamination was confirmed by using the RNA as a template in standard PCR to amplify fragments of the glyceraldehyde-3-phosphate dehydrogenase (*GAPDH*) gene. To prevent degradation, all RNAs were stored at −80 °C until further use.

### Expression microarray

500 ng of total RNA was reverse-transcribed into cDNA with the Ambion WT Expression Kit (Life Technologies GmbH, Darmstadt, Germany). Biotin-labeled cRNA targets were made using the Affymetrix GeneChip WT Terminal Labeling Kit (Affymetrix, Santa Clara, CA, USA). Fragmented biotin-labeled cRNAs were hybridized onto Chicken Gene 1.0 ST Arrays (Affymetrix), which contains 18,214 probe-sets. After staining and washing, the arrays were scanned and raw data were obtained with the Affymetrix GCOS 1.1.1 software.

### Normalization and statistical analysis

For expression data analysis raw data (cel-files) obtained by Affymetrix GCOS 1.1.1 software of all arrays were used as input files for the Affymetrix Expression Console for subsequent normalization and estimation of expression levels. Quantitative expression levels of transcripts were estimated using PLIER algorithm (Probe Logarithmic Intensity Error) and using DABG (detection above background) to evaluate detection by combining probe-level *p*-values to generate probe cell intensity values at exon level. All data were deposited in a MIAME-compliant database, the National Center for Biotechnology Information Gene Expression Omnibus (www.ncbi.nlm.nih.gov/geo; accession number: GSE76670). All “present” values (default settings with detection *p*-values of ≤ 0.04) were selected and integrated within gene-level annotation. To extract the outlying and nonspecific results, criteria on standard deviation (SD ≥ 0.16) and means (m ≥ 2.5) were applied using “genefilter” in R (www.r-project.org). Changes in transcript abundance were determined by analysis of variance (JMP Genomics, SAS-Institute) considering individual and combined effects of temperature, treatment period and gender and slaughter weight. Sex was excluded from the statistical model due to marginal effects. The final model included fixed effects of temperature, treatment period and interactions. Slaughter weight was included as covariate for the 35 days post-hatched time course. Comparisons of treated samples (L and H) to controls (C) within the respective time points (E10, E13, D35) were considered. Transcripts with significant differences of abundance at *p*-values ≤ 0.05 were selected and queried for pathways analysis. At *pre-hatch* stages *p* ≤ 0.05 equals FDR adjusted *p*-values of q ≤ 0.18; at D35 *p* ≤ 0.05 corresponding q-values ranged between 0.35 and 0.70.

### Real time quantitative RT-PCR (qPCR)

For validation of microarray data, the gene expression of three genes was determined by Real-time quantitative PCRs using the same D35 samples used for microarray analyses. The assays were done in duplicate in volumes of 10 μl using the LightCycler 480 SYBR Green I Master Kit (Roche), on a LightCycler 480 Real-Time PCR System (Roche Diagnostics GmbH, Germany). The temperature profiles comprised an initial denaturation step at 95 °C for 10’ and 40 cycles consisting of denaturation at 95 °C for 15”, annealing at 60 °C for 10” and extension at 72 °C for 15”. The amplified genes were *GAPH* and *ACTP* as well as *FGA*, *NR43A* and *AHSG* (Additional file 7), where the first two were used as reference genes to account for variation of cDNA amounts after reverse transcription by calculating a normalization factor. Target genes were selected because of their redundant assignment to different but related biofunctions. For all the assays threshold cycles were converted to copy numbers using a standard curve generated by amplifying serial dilutions of an external PCR standard (10^7^ - 10^2^ copies). After completion of amplification protocol all samples were subjected to melting curve analyses and gel electrophoresis. Primers were obtained from Sigma-Aldrich, Germany.

### Pathway analyses and major categories

Least-squares means of expression level and fold changes including “UP” and “DOWN” regulation among the tissues were estimated. Annotation data for Affymetrix Chicken Genome Arrays were obtained from the producer (Affymetrix Chicken Genome Array annotations release 34). Ingenuity Pathway Analysis was used for functional annotation estimation of association between dataset and pathway. Differentially expressed genes (DEGs) were analyzed referring to Ingenuity Pathways Knowledge Base (IPKB). Biological and canonical pathways were identified from the IPKB library. Significance was considered based on Fisher’s exact test *p*-values adjusted for multiple testing using the Benjamini-Hochberg correction procedure. Cut-off criteria were set to corrected BH *p*-values ≤ 0.05 for canonical pathways and for biofunctions, respectively. The variation of pathways was assigned, and we focused on the top most affected biological functions related to tissue development and myogenesis. All pathways were grouped into new categories based on criteria concerning the major roles in comprehensive biological routes on organismal, organ, tissue, cell or molecular levels. All biological functions were categorized in eight major groups (gr. 1 – gr. 8) as follows: cell maintenance, proliferation, differentiation, and replacement (gr. 1); organismal organ and tissue development (gr. 2); nutrient metabolism (gr. 3); genetic information and nucleic acid processing (gr. 4); molecular transport (gr. 5); cell signaling and interaction (gr. 6); small molecule biochemistry (gr. 7); and response to stimuli (gr. 8). “Activated” and “deactivated” genes were assigned by positive and negative Z-scores, predicting the activation state of related transcription regulators. Significant pathways that were altered with *in-ovo* temperature modifications were clustered and visualized by heatmap. Genes assigned to major categories, as defined below, and with Z-scores were selected to derive IPA networks. Top network results were displayed covering related DEGs with annotation from NCBI reference sequence base [54].

### Availability of supporting data

All supporting data are included in additional files.

## Additional files

Additional file 1:
**Table S1.** Common DEGs at embryonic stages and D35. **Table S2.** Common DEGs of H10 and H13 or L10 and L13 at embryonic stages. **Table S3.** Common DEGs of H10 and H13 or L10 and L13 at D35. (DOCX 41 kb) Additional file 2: Assignment of DEGs to major categories, and biological functions obtained at embryonic stage for early treatment; H10UΔC, H10DΔC, L10UΔC and L10DΔC. (DOCX 21 kb) Additional file 3: Assignment of DEGs to major categories, and biological functions obtained at embryonic stage for late treatment; H13UΔC, H13DΔC, L13UΔC and L13DΔC. (DOCX 23 kb) Additional file 4: Assignment of DEGs to major categories, and biological functions obtained at D35 for early treatment; H10UΔC, H10DΔC, L10UΔC and L10DΔC (DOCX 22 kb) Additional file 5: Assignment of DEGs to major categories, and biological functions obtained at D35 for late treatment; H13UΔC, H13DΔC, L13UΔC and L13DΔC. (DOCX 23 kb) Additional file 6: Body weight, carcass weight and weight of hind muscles of broilers of the experimental groups used for expression analyses. (DOCX 19 kb) Additional file 7: Primers used for quantitative real-time PCR (qPCR) (DOCX 15 kb)

## Abbreviations

ΔC: ‘delta’ control, difference treatment versus control
D35: post-hatch days 35, slaughter date
DEGs: differentially expressed genes
E7, E10, E13: embryonic days 7^th^, 10^th^ and 13^th^ respectively
gr.1: major category group 1 cell maintenance, proliferation, differentiation and replacement
gr.2: major category group 2 organismal, organ, and tissue development
gr.3: major category group 3 nutrient metabolism
gr.4: major category group 4 genetic information and nucleic acid processing
gr.5: major category group 5 molecular transport
gr.6: major category group 6 cell signaling and interaction
gr.7: major category group 7 small molecule biochemistry
gr.8: major category group 8 response to stimuli
H10: embryos were incubated at high temperature (38.8 °C) at embryonic days 7–10 and at control temperature (37.8 °C) at remaining time before and after
H13: embryos were incubated at high temperature (38.8 °C) at embryonic days 10–13 and at control temperature (37.8 °C) at remaining time before and after
IPA: Ingenuity Pathway Analysis
L10: embryos were incubated at low temperature (36.8 °C) at embryonic days 7–10 and at control temperature (37.8 °C) at remaining time before and after
L13: embryos were incubated at low temperature (36.8 °C) at embryonic days 10–13 and at control temperature (37.8 °C) at remaining time before and after
