# Supplementary material for: Immediate and long-term transcriptional response of hind muscle tissue to transient variation of incubation temperature in broilers
Source: BMC Genomics. 2016 May 4;17:323. doi: 10.1186/s12864-016-2671-9 (PMC4855815; doi:10.1186/s12864-016-2671-9)
Supplement: Additional file 1: — Table S1. Common DEGs at embryonic stages and D35. Table S2. Common DEGs of H10 and H13 or L10 and L13 at embryonic stages. Table S3. Common DEGs of H10 and H13 or L10 and L13 at D35. (DOCX 41 kb) [file 12864_2016_2671_MOESM1_ESM.docx]

**Additional file 1**

**Table 1** Common DEGs at embryonic stages and D35.

| Group | Transcript id | Gene symbol | Embryo | | | Mature | | |
| --- | --- | --- | --- | --- | --- | --- | --- | --- |
|  |  |  | P-value | FC* | FC trend | P-value | FC* | FC trend |
| H10 | 15393184 | HDHD1A | 0.03 | 1.33 | UP | 0.04 | -1.04 | DOWN |
|  | 15415679 | ARYL_CHICK | 0.01 | 1.71 | UP | 0.04 | -1.13 | DOWN |
|  | 15429749 | SHMT1 | 0.02 | 1.63 | UP | 0.03 | -1.14 | DOWN |
|  | 15444897 | CCDC55 | <0.01 | 1.71 | UP | <0.01 | -1.30 | DOWN |
|  | 15449899 | TSPAN13 | 0.01 | 1.52 | UP | 0.04 | -1.04 | DOWN |
|  | 15475100 | CCDC153 | 0.03 | -1.16 | DOWN | 0.01 | 1.13 | UP |
|  | 15486194 | ETAA1 | 0.04 | 1.41 | UP | 0.05 | -1.10 | DOWN |
|  | 15490320 | DDO | 0.01 | 1.65 | UP | 0.02 | -1.10 | DOWN |
|  | 15499195 | ADI1 | 0.03 | 1.25 | UP | 0.01 | -1.04 | DOWN |
|  | 15505748 | Q60GU0_CHICK | <0.01 | 1.53 | UP | <0.01 | -1.09 | DOWN |
|  | 15506087 | KIAA0232 | 0.02 | 1.31 | UP | 0.02 | -1.05 | DOWN |
|  | 15511713 | Q5ZLP7_CHICK | 0.04 | 1.38 | UP | 0.02 | -1.09 | DOWN |
|  | 15517860 | ZNF839 | 0.04 | 1.30 | UP | 0.02 | -1.10 | DOWN |
|  | 15541032 | HOOK1_CHICK | 0.02 | 1.57 | UP | 0.02 | -1.12 | DOWN |
|  | 15545357 | KLHL24 | 0.01 | 1.18 | UP | 0.01 | -1.08 | DOWN |
|  | 15551431 | MYH11 | <0.01 | 2.23 | UP | 0.05 | -1.09 | DOWN |
|  | 15560998 | MCCC2 | 0.02 | 1.41 | UP | 0.03 | -1.10 | DOWN |
| L10 | 15418759 | TMEM111 | 0.04 | 1.22 | UP | 0.02 | -1.09 | DOWN |
|  | 15435883 | MTFP1 | 0.05 | 1.23 | UP | 0.04 | -1.11 | DOWN |
|  | 15458689 | Q9DDD4_CHICK | <0.01 | -1.29 | DOWN | 0.04 | -1.06 | DOWN |
|  | 15491144 | IBTK | 0.03 | -1.33 | DOWN | 0.01 | -1.05 | DOWN |
|  | 15494686 | MIA3 | 0.04 | -1.23 | DOWN | 0.03 | -1.03 | DOWN |
|  | 15532933 | SP3_CHICK | 0.04 | -1.23 | DOWN | 0.02 | -1.04 | DOWN |
|  | 15535607 | SDPR | 0.02 | -1.18 | DOWN | 0.03 | -1.03 | DOWN |
|  | 15535718 | C2orf66 | 0.04 | -1.45 | DOWN | 0.03 | -1.15 | DOWN |
|  | 15554202 | MANSC1 | 0.04 | -1.38 | DOWN | 0.01 | -1.07 | DOWN |
| H13 | 15403705 | RN5-8S1 | 0.01 | -1.24 | DOWN | 0.03 | -1.02 | DOWN |
|  | 15434604 | Q5ZKS2_CHICK | 0.05 | 1.28 | UP | 0.05 | -1.09 | DOWN |
|  | 15436224 | CLEC2B | <0.01 | 5.28 | UP | 0.01 | 1.18 | UP |
|  | 15436422 | CLEC2B | 0.03 | 3.75 | UP | 0.04 | 1.34 | UP |
|  | 15436477 | MR1 | 0.01 | 2.82 | UP | 0.02 | 1.19 | UP |
|  | 15436481 | SBF1 | 0.02 | 1.99 | UP | 0.02 | 1.05 | UP |
|  | 15459552 | KCNG2_CHICK | 0.02 | 1.28 | UP | 0.03 | -1.10 | DOWN |
|  | 15463264 | ABRA | 0.05 | 1.49 | UP | 0.04 | -1.06 | DOWN |
|  | 15483075 | SNORD37 | 0.01 | -1.70 | DOWN | 0.01 | -1.14 | DOWN |
|  | 15509009 | IL15 | 0.01 | 1.46 | UP | 0.03 | -1.09 | DOWN |
|  | 15551255 | RN5-8S1 | 0.01 | -1.24 | DOWN | 0.03 | -1.02 | DOWN |
|  | 15552380 | BAHCC1 | 0.04 | 1.14 | UP | 0.01 | -1.05 | DOWN |
| L13 | 15385410 | PANX2 | 0.05 | -1.62 | DOWN | 0.05 | -1.60 | DOWN |
|  | 15412168 | Q5ZLD9_CHICK | 0.02 | 1.19 | UP | 0.04 | -1.02 | DOWN |
|  | 15425500 | SAP30L | 0.01 | 1.15 | UP | 0.03 | -1.02 | DOWN |
|  | 15439176 | SLC25A25 | 0.03 | 1.37 | UP | 0.05 | -1.06 | DOWN |
|  | 15442540 | C17orf109 | 0.05 | -1.31 | DOWN | 0.04 | 1.05 | UP |
|  | 15447772 | Q5ZHK3_CHICK | 0.01 | 1.38 | UP | 0.05 | -1.03 | DOWN |
|  | 15448513 | ACTR3B | 0.02 | 1.63 | UP | 0.01 | -1.02 | DOWN |
|  | 15455056 | BAALC | <0.01 | -1.28 | DOWN | 0.01 | 1.14 | UP |
|  | 15457355 | TFPI2 | 0.03 | -1.36 | DOWN | 0.05 | 1.05 | UP |
|  | 15474591 | MYOM3 | 0.04 | -1.90 | DOWN | 0.01 | 1.26 | UP |
|  | 15500862 | FUNDC2 | 0.04 | 1.26 | UP | 0.01 | 1.02 | UP |
|  | 15533532 | CHPF | 0.03 | 1.41 | UP | 0.03 | -1.01 | DOWN |
|  | 15538781 | MIR214 | 0.04 | -1.20 | DOWN | 0.03 | -1.18 | DOWN |
|  | 15543488 | Q6ZYP0_CHICK | 0.04 | -1.38 | DOWN | 0.03 | -1.08 | DOWN |
|  | 15547330 | SLITRK3 | 0.02 | -1.25 | DOWN | 0.01 | 1.09 | UP |
|  | 15552396 | SLMO1 | 0.03 | 1.35 | UP | 0.03 | -1.05 | DOWN |
|  | 15556394 | OR37 | 0.03 | -1.45 | DOWN | 0.04 | -1.52 | DOWN |

*Fold change (FC) for each gene was calculated by using the ratio of least square means on the original scale.

**Table 2** Common DEGs of H10 and H13 or L10 and L13 at embryonic stages.

| Treatment | Transcript id | Gene symbol | ED7-10 | | | ED10-13 | | |
| --- | --- | --- | --- | --- | --- | --- | --- | --- |
|  |  |  | P-value | FDR-adj. P-value | FC* | P-value | FDR-adj. P-value | FC* |
| High  (38.8 °C) | 15385719 | TMEM168 | 0.01 | 0.06 | 1.16 | 0.04 | 0.17 | 1.12 |
|  | 15389727 | TTC38 | 0.04 | 0.16 | 1.36 | 0.05 | 0.17 | 1.35 |
|  | 15405625 | AFF3 | 0.02 | 0.10 | -1.29 | 0.02 | 0.10 | 1.29 |
|  | 15405640 | AFF3 | <0.01 | 0.02 | -1.30 | <0.01 | 0.03 | 1.29 |
|  | 15439952 | PDCL | 0.04 | 0.14 | 1.16 | 0.05 | 0.17 | 1.15 |
|  | 15453399 | METTL4 | <0.01 | 0.02 | 1.34 | <0.01 | 0.03 | 1.31 |
|  | 15421109 | mir-1 | <0.01 | 0.02 | 2.12 | 0.02 | 0.09 | 1.80 |
|  | 15483075 | SNORD37 | <0.01 | 0.03 | -1.72 | 0.01 | 0.04 | -1.70 |
|  | 15500752 | FRMPD3 | 0.03 | 0.13 | 1.18 | 0.04 | 0.16 | 1.17 |
|  | 15509009 | IL15 | 0.02 | 0.11 | 1.37 | 0.01 | 0.05 | 1.46 |
|  | 15511061 | RG9MTD2 | 0.04 | 0.15 | 1.19 | 0.04 | 0.15 | 1.19 |
|  | 15522960 | THSD3 | 0.01 | 0.06 | -1.13 | 0.03 | 0.12 | -1.11 |
|  | 15546236 | AGTR1 | <0.01 | <0.01 | 1.46 | 0.01 | 0.05 | 1.30 |
|  | 15463264 | ABRA | <0.01 | 0.03 | 1.84 | 0.05 | 0.18 | 1.49 |
|  | 15521222 | CRY2_CHICK | 0.02 | 0.08 | 1.13 | <0.01 | 0.01 | 1.19 |
|  | 15406211 | CLYBL | <0.01 | 0.02 | 1.36 | <0.01 | 0.03 | 1.34 |
|  | 15527600 | CASP7 | <0.01 | 0.02 | 1.55 | 0.03 | 0.13 | 1.36 |
| Low  (36.8 °C) | 15387312 | H1_CHICK | 0.02 | 0.08 | 1.65 | <0.01 | 0.02 | -1.90 |
|  | 15400863 | MYBPC1 | 0.05 | 0.18 | -1.22 | 0.03 | 0.14 | -1.24 |
|  | 15431552 | YPEL1 | 0.01 | 0.08 | -1.23 | <0.01 | 0.03 | -1.27 |
|  | 15453020 | CD226 | 0.03 | 0.13 | -1.24 | 0.01 | 0.07 | -1.29 |
|  | 15473494 | PNRC2_CHICK | 0.04 | 0.16 | -1.22 | 0.04 | 0.15 | -1.23 |
|  | 15500611 | Gm5127 | 0.01 | 0.04 | 1.24 | 0.03 | 0.12 | -1.18 |
|  | 15544538 | FGGY | 0.04 | 0.14 | -1.20 | 0.05 | 0.18 | -1.18 |
|  | 15550889 | LOC100653240 | 0.03 | 0.13 | 1.26 | 0.01 | 0.05 | 1.34 |
|  | 15552699 | HNRNPK | <0.01 | 0.02 | -3.01 | 0.03 | 0.12 | -2.11 |
|  | 15548143 | LRRC33 | 0.03 | 0.13 | 1.15 | 0.03 | 0.12 | 1.16 |

*Fold change (FC) for each gene was calculated by using the ratio of least square means on the original scale.

**Table 3** Common DEGs of H10 and H13 or L10 and L13 at D35.

| Treatment | Transcript id | Gene symbol | ED7-10 | | | ED10-13 | | |
| --- | --- | --- | --- | --- | --- | --- | --- | --- |
|  |  |  | P-value | FDR-adj. P-value | FC* | P-value | FDR-adj. P-value | FC* |
| High  (38.8 °C) | 15418520 | GgaAffx.1460.1.S1_at | <0.01 | 0.36 | -1.06 | 0.05 | 0.69 | 1.03 |
|  | 15420113 | C3orf14 | 0.03 | 0.63 | -1.11 | 0.01 | 0.44 | 1.10 |
|  | 15425244 | FAM193B | 0.03 | 0.64 | -1.11 | 0.04 | 0.68 | 1.07 |
|  | 15429749 | SHMT1 | 0.03 | 0.65 | -1.14 | <0.01 | 0.38 | -1.14 |
|  | 15437904 | NOE1_CHICK | 0.01 | 0.50 | -1.17 | 0.02 | 0.60 | 1.11 |
|  | 15506087 | KIAA0232 | 0.02 | 0.61 | -1.05 | 0.03 | 0.63 | 1.04 |
|  | 15518609 | Q5ZJ03_CHICK | 0.05 | 0.70 | 1.06 | <0.01 | 0.35 | -1.07 |
|  | 15522880 | ZDHHC22 | 0.05 | 0.69 | -1.14 | 0.04 | 0.67 | -1.10 |
|  | 15545355 | GPR148 | 0.05 | 0.69 | 1.22 | 0.01 | 0.45 | -1.18 |
| Low  (36.8 °C) | 15385427 | SELO | 0.02 | 0.59 | -1.06 | 0.04 | 0.66 | -1.03 |
|  | 15392435 | MIR221 | 0.03 | 0.63 | 1.10 | 0.01 | 0.56 | 1.07 |
|  | 15400955 | JHDM1D | 0.03 | 0.63 | -1.03 | 0.04 | 0.67 | -1.02 |
|  | 15401885 | Q5ZKY8_CHICK | 0.01 | 0.45 | -1.11 | <0.01 | 0.35 | -1.09 |
|  | 15402320 | RM51_CHICK | 0.02 | 0.57 | -1.05 | 0.03 | 0.63 | -1.03 |
|  | 15413556 | IGDCC4 | 0.02 | 0.57 | -1.08 | 0.01 | 0.55 | -1.06 |
|  | 15417660 | Q5ZKH7_CHICK | <0.01 | 0.41 | -1.04 | 0.03 | 0.64 | -1.02 |
|  | 15417733 | SPATA2L | 0.03 | 0.65 | -1.03 | 0.03 | 0.65 | -1.02 |
|  | 15418520 | GgaAffx.1460.1.S1_at | <0.01 | 0.37 | -1.06 | 0.03 | 0.65 | 1.03 |
|  | 15421138 | TIMP4 | 0.04 | 0.68 | -1.08 | 0.04 | 0.67 | 1.06 |
|  | 15421147 | RAF1_CHICK | 0.02 | 0.57 | -1.03 | 0.03 | 0.66 | -1.02 |
|  | 15424161 | VDAC1 | 0.01 | 0.49 | -1.02 | 0.05 | 0.69 | -1.01 |
|  | 15429603 | DHRS7B | 0.04 | 0.67 | -1.04 | 0.03 | 0.64 | 1.03 |
|  | 15431766 | TMEM132D | 0.02 | 0.60 | 2.00 | 0.01 | 0.55 | -1.44 |
|  | 15435883 | MTFP1 | 0.04 | 0.68 | -1.11 | 0.03 | 0.66 | 1.08 |
|  | 15440960 | MYADML2 | 0.04 | 0.68 | -1.04 | 0.03 | 0.62 | 1.03 |
|  | 15445815 | PITPNM3 | 0.02 | 0.57 | -1.04 | <0.01 | 0.19 | -1.05 |
|  | 15445837 | FBXO39 | 0.04 | 0.68 | -1.12 | 0.01 | 0.47 | -1.12 |
|  | 15455056 | BAALC | 0.04 | 0.68 | 1.18 | 0.01 | 0.53 | 1.14 |
|  | 15455419 | MYC_CHICK | 0.01 | 0.49 | -1.12 | 0.04 | 0.68 | 1.06 |
|  | 15455798 | SEC22C | 0.01 | 0.53 | -1.09 | 0.05 | 0.69 | -1.05 |
|  | 15470717 | VPS13D | 0.03 | 0.66 | -1.04 | 0.02 | 0.59 | -1.03 |
|  | 15473220 | KHDR1_CHICK | 0.04 | 0.66 | -1.05 | 0.03 | 0.63 | -1.04 |
|  | 15474584 | FABP1 | <0.01 | 0.44 | 1.63 | 0.02 | 0.60 | 1.25 |
|  | 15479868 | CD34 | 0.01 | 0.46 | -1.08 | <0.01 | 0.43 | -1.06 |
|  | 15494525 | GPCPD1 | 0.02 | 0.57 | -1.08 | 0.05 | 0.69 | -1.05 |
|  | 15505907 | FGFBP1 | 0.03 | 0.63 | 1.21 | <0.01 | 0.19 | 1.23 |
|  | 15506701 | LAS1L | 0.01 | 0.51 | -1.06 | 0.02 | 0.60 | -1.04 |
|  | 15510785 | SYNPO2 | 0.01 | 0.54 | -1.02 | 0.03 | 0.64 | -1.01 |
|  | 15517721 | C14orf68 | 0.01 | 0.49 | 1.20 | 0.03 | 0.63 | 1.10 |
|  | 15517863 | TECPR2 | 0.02 | 0.61 | -1.04 | 0.02 | 0.58 | -1.03 |
|  | 15518132 | ADSSL1 | 0.01 | 0.46 | -1.06 | 0.04 | 0.68 | -1.03 |
|  | 15519323 | LGR4 | 0.03 | 0.64 | -1.06 | 0.05 | 0.68 | -1.04 |
|  | 15521663 | UBR1 | 0.01 | 0.53 | -1.05 | 0.05 | 0.69 | -1.03 |
|  | 15523533 | ATG2B | 0.03 | 0.65 | -1.03 | 0.01 | 0.48 | -1.02 |
|  | 15529325 | MYST4 | 0.01 | 0.47 | -1.06 | 0.02 | 0.59 | -1.04 |
|  | 15532138 | COQ10B | 0.03 | 0.63 | -1.04 | 0.04 | 0.68 | -1.03 |
|  | 15533532 | CHPF | <0.01 | 0.22 | -1.03 | 0.03 | 0.65 | -1.01 |
|  | 15541808 | CDC73_CHICK | 0.03 | 0.64 | -1.03 | 0.02 | 0.60 | -1.02 |
|  | 15542055 | PPAPDC2 | 0.01 | 0.54 | -1.06 | 0.04 | 0.67 | -1.04 |
|  | 15545355 | GPR148 | <0.01 | 0.40 | 1.34 | 0.03 | 0.63 | -1.14 |
|  | 15546260 | Q5G8Y9_CHICK | 0.05 | 0.69 | -1.13 | 0.01 | 0.54 | 1.11 |
|  | 15550074 | Q67BJ2_CHICK | 0.04 | 0.67 | -1.11 | 0.04 | 0.67 | -1.07 |
|  | 15552396 | SLMO1 | 0.04 | 0.67 | -1.07 | 0.03 | 0.65 | -1.05 |
|  | 15554202 | MANSC1 | 0.01 | 0.47 | -1.07 | 0.02 | 0.56 | -1.04 |

*Fold change (FC) for each gene was calculated by using the ratio of least square means on the original scale.
