# Supplementary material for: Immediate and long-term transcriptional response of hind muscle tissue to transient variation of incubation temperature in broilers
Source: BMC Genomics. 2016 May 4;17:323. doi: 10.1186/s12864-016-2671-9 (PMC4855815; doi:10.1186/s12864-016-2671-9)
Supplement: Additional file 2: — Assignment of DEGs to major categories, and biological functions obtained at embryonic stage for early treatment; H10UΔC, H10DΔC, L10UΔC and L10DΔC. (DOCX 21 kb) [file 12864_2016_2671_MOESM2_ESM.docx]

**Additional file 2:** Assignment of DEGs to major categories, and biological functions obtained at embryonic stage for early treatment; H10UΔC, H10DΔC, L10UΔC and L10DΔC.

| **Major category** | **Ratio*** | **Biological function** | **BH P-value** | **Z-score** | **Total DEGs** | **DEGs assigned to biofunction**** |
| --- | --- | --- | --- | --- | --- | --- |
| **H10UΔC** |  |  |  |  |  |  |
| **Cell maintenance, proliferation differentiation and replacement** |  | Structure of sarcomere | 1.64E-02 |  | 3 | DICER1, NEB, PPARGC1A |
| **Organismal, organ and tissue development** | 1:1 | Mass of epigonadal fat pad | 3.60E-02 | -0.478 | 7 | CEBPB, GHR, GPAM, IGF1, IL1R1, INSIG1, PPARA |
|  |  | Percentage body fat | 3.54E-02 | 0.152 | 10 | ADRA1B, AGPAT2, AKT1, CEBPB, COMT, GHR, GPAM, IL1R1, PPARA, PPARGC1A |
| **Nutrient metabolism** | 4:0 | Concentration of fatty acid | 2.57E-02 | 0.32 | 23 | PPARA, AGPAT2, AHSG, AKT1, CEBPA, CEBPB, CIDEA, ECI1, FABP1, FGF7 |
|  |  | Concentration of acylglycerol | 2.57E-02 | 0.499 | 27 |  |
|  |  | Concentration of triacylglycerol | 4.79E-02 | 0.931 | 24 |  |
|  |  | Metabolism of amino acids | 2.57E-02 | 1.951 | 16 |  |
| **Cell signaling and interaction** |  | Insulin-like growth factor receptor signaling pathway | 1.67E-02 |  | 5 | ADRA1B, AGTR1, AKT1, ARHGAP29, ARHGAP5, ARHGEF3, DLC1, GHR, IGF1, PIK3R1 |
|  |  | Rho protein signal transduction | 4.23E-02 |  | 9 |  |
| **Small molecule biochemistry** | 4:0 | Beta-oxidation of lipid | 2.10E-05 | 1.72 | 15 | ABCD3, ACADSB, ACOX2, ACOX3, BDH2, CPT1A, CPT2, DECR1, ECHS1, ECI1 |
|  |  | Oxidation of lipid | 2.92E-05 | 2.055 | 27 |  |
|  |  | Oxidation of fatty acid | 4.18E-05 | 2.133 | 22 |  |
|  |  | Beta-oxidation of fatty acid | 4.18E-05 | 2.355 | 14 |  |
| **H10DΔC** |  |  |  |  |  |  |
| **Organismal, organ and tissue development** |  | Development of joint | 7.10E-04 |  | 4 | NFATC1, NFATC2, FMOD, NOG |
|  |  | Formation of osteophyte | 3.17E-02 |  | 2 |  |
| **L10UΔC** |  |  |  |  |  |  |
| **Cell maintenance, proliferation differentiation and replacement** |  | Production of lymphocytes | 2.34E-02 |  | 2 | ETV6, LIMS1, NFKBIA, CNR2, WNT11 |
|  |  | Depletion of marginal-zone B lymphocytes | 2.34E-02 |  | 1 |  |
|  |  | Apoptosis of embryonic cells | 2.80E-02 |  | 2 |  |
|  |  | Apoptosis of breast cell lines | 3.14E-02 |  | 2 |  |
|  |  | Maturation of fibroblast cell lines | 3.41E-02 |  | 1 |  |
| **Organismal, organ and tissue development** |  | Aggregation of intestinal cell lines | 2.34E-02 |  | 1 | CNR2, ETV6, LIMS1, NFKBIA, WNT11 |
|  |  | Delay in initiation of branching of mammary duct | 2.34E-02 |  | 1 |  |
|  |  | Expansion of cortical bone | 2.34E-02 |  | 1 |  |
|  |  | Quantity of colony forming unit myeloid cell | 2.80E-02 |  | 1 |  |
|  |  | Function of myocardium | 3.41E-02 |  | 1 |  |
| **Nutrient metabolism** |  | Phosphorylation of D-pantothenic acid | 2.34E-02 |  | 1 | ENTPD2, GLTP, NFKBIA, PANK2, PANK2 |
|  |  | Conversion of ADP | 2.34E-02 |  | 1 |  |
|  |  | Transport of glycolipid | 3.90E-02 |  | 1 |  |
|  |  | Synthesis of 12(S)-hydroxyeicosatetraenoic acid | 4.24E-02 |  | 1 |  |
|  |  | Synthesis of coenzyme A | 4.46E-02 |  | 1 |  |
| **Genetic information and nucleic acid processing** |  | Catabolism of purine ribonucleoside diphosphate | 3.41E-02 |  | 1 | ENTPD2, ETV6 |
|  |  | Annealing of DNA | 4.46E-02 |  | 1 |  |
|  |  | Binding of Ets element | 4.80E-02 |  | 1 |  |
| **Molecular transport** |  | Release of nitrite | 4.24E-02 |  | 1 |  |
|  |  | Transport of lactic acid | 4.24E-02 |  | 1 |  |
| **Cell signaling and interaction** |  | Recruitment of antigen presenting cells | 4.30E-02 |  | 2 | CNR2, NFKBIA |
|  |  | Binding of nervous tissue cell lines | 4.05E-02 |  | 1 |  |
| **Small molecule biochemistry** |  | Conversion of ATP | 4.05E-02 |  | 1 |  |
| **Response to stimuli** |  | Relocalization of marginal-zone B lymphocytes | 2.80E-02 |  | 1 | CNR2, ETV6, NFKBIA |
|  |  | Invasion of T lymphocytes | 3.90E-02 |  | 1 |  |
|  |  | Inflammation of synovial membrane | 4.05E-02 |  | 1 |  |
|  |  | Inflammation of interstitial tissue | 4.31E-02 |  | 1 |  |
| **L10DΔC** |  |  |  |  |  |  |
| **Organismal, organ and tissue development** |  | Cardiogenesis | 1.34E-02 |  | 10 | CASP7, CHD7, HIF1A, PTPN11, REST, SEMA3C, SMARCD3, TTN, USP8, XIRP1 |

* between positive and negative Z-score

**at maximum 10 genes are shown
