# Supplementary material for: Immediate and long-term transcriptional response of hind muscle tissue to transient variation of incubation temperature in broilers
Source: BMC Genomics. 2016 May 4;17:323. doi: 10.1186/s12864-016-2671-9 (PMC4855815; doi:10.1186/s12864-016-2671-9)
Supplement: Additional file 3: — Assignment of DEGs to major categories, and biological functions obtained at embryonic stage for late treatment; H13UΔC, H13DΔC, L13UΔC and L13DΔC. (DOCX 23 kb) [file 12864_2016_2671_MOESM3_ESM.docx]

**Additional file 3:** Assignment of DEGs to major categories, and biological functions obtained at embryonic stage for late treatment; H13UΔC, H13DΔC, L13UΔC and L13DΔC.

| **Major category** | **Ratio*** | **Biological function** | **BH P-value** | **Z-score** | **Total DEGs** | **DEGs assigned to biofunction**** |
| --- | --- | --- | --- | --- | --- | --- |
| **H13UΔC** |  |  |  |  |  |  |
| **Cell maintenance, proliferation differentiation**  **And replacement** |  | Autophagy of Cytoplasm | 4.58E-02 |  | 2 | TLR4, TLR6, PLAT, CD74, EGR1, LRP1, mir-15, NEURL1 |
|  |  | Function of antigen presenting cells | 4.58E-02 |  | 5 |  |
|  |  | Cell death of Schwann cells | 4.58E-02 |  | 2 |  |
|  |  | Apoptosis of pancreatic stellate cells | 4.58E-02 |  | 1 |  |
|  |  | Movement of sperm tail | 4.58E-02 |  | 1 |  |
| **Organismal, organ and tissue development** |  | Structural integrity of basement membrane | 1.83E-02 |  | 2 | COL4A1, KCNA4, PLAT, VEGFC |
|  |  | Angiogenesis of epithelial tissue | 4.58E-02 |  | 2 |  |
|  |  | Activation of ventricular myocytes | 4.58E-02 |  | 1 |  |
|  |  | Area of lymphoid tissue | 4.58E-02 |  | 1 |  |
|  |  | Expansion of vein | 4.58E-02 |  | 1 |  |
| **Nutrient metabolism** |  | Recognition of hyaluronic acid | 4.58E-02 |  | 1 | TLR4, AKR1B1, AKT3, EGR1, ENTPD5, FKBP4, LRP1, PLAT, PLOD1, TLR6 |
|  |  | Quantity of steroid | 4.58E-02 |  | 7 |  |
|  |  | Recognition of lipid | 4.58E-02 |  | 2 |  |
|  |  | Biosynthesis of hydroxylysine | 4.58E-02 |  | 1 |  |
|  |  | Biosynthesis of sorbitol | 4.58E-02 |  | 1 |  |
| **Genetic information and nucleic acid processing** |  | Hydroxylation of protein fragment | 4.58E-02 |  | 1 | PLOD1 |
| **Cell signaling and interaction** |  | Binding of colon cell lines | 4.58E-02 |  | 1 | TLR4, LRP1 |
|  |  | Binding of colonocytes | 4.58E-02 |  | 1 |  |
|  |  | Accumulation of focal adhesions | 4.58E-02 |  | 1 |  |
|  |  | Attachment of carcinoma cell lines | 4.58E-02 |  | 1 |  |
|  |  | Detachment of carcinoma cell lines | 4.58E-02 |  | 1 |  |
| **Small molecule biochemistry** |  | Degradation of histamine | 4.58E-02 |  | 1 | HNMT |
| **Response to stimuli** |  | Antigen presentation | 4.58E-02 |  | 3 | TLR4, CD1B, CD74 |
|  |  | Clustering of B lymphocytes | 4.58E-02 |  | 1 |  |
|  |  | Acute inflammatory response of airway | 4.58E-02 |  | 1 |  |
| **H13DΔC** |  |  |  |  |  |  |
| **Cell maintenance, proliferation differentiation and replacement** | 0:2 | Invasion of cells | 3.03E-02 | -1.033 | 4 | ANG, mir-17, ADAM21, ARHGAP18, CNP, mir-146 |
|  |  | Development of cytoplasm | 2.54E-02 | -0.914 | 4 |  |
| **Organismal, organ and tissue development** | 2:2 | Development of blood vessel | 2.54E-02 | -0.406 | 5 | ANG, CX3CR1, mir-146, mir-17, DLX3 |
|  |  | Vasculogenesis | 2.99E-02 | -0.13 | 4 |  |
|  |  | Quantity of lymphocytes | 3.03E-02 | 0.258 | 4 | mir-146, mir-17, RAG2, CX3CR1, CNP, CX3CR1, DLX3, KCNA1 |
|  |  | Organismal death | 4.02E-02 | 2.166 | 7 |  |
| **Nutrient metabolism** |  | Induction of cyclic GMP | 2.99E-02 |  | 1 | CNP |
| **Genetic information and nucleic acid processing** |  | Rearrangement of gene | 2.94E-02 |  | 1 | ANG, RAG2 |
|  |  | Cleavage of trna | 2.99E-02 |  | 1 |  |
|  |  | Cleavage of RNA fragment | 3.57E-02 |  | 1 |  |
|  |  | Cleavage of DNA fragment | 3.84E-02 |  | 1 |  |
|  |  | Transcription of rrna | 3.90E-02 |  | 1 |  |
| **Cell signaling and interaction** |  | Activation of cells | 3.90E-02 | 0.548 | 4 | ANG, CX3CR1, mir-146, mir-17 |
| **Response to stimuli** |  | Inflammation of organ | 3.42E-02 | 1.982 | 5 | CX3CR1, KCNA1, mir-146, mir-17, RAG2 |
| **L13UΔC** |  |  |  |  |  |  |
| **Cell maintenance, proliferation differentiation and replacement** | 0:3 | Apoptosis | 2.43E-02 | -1.569 | 54 | ATG16L1, ATXN3, B2M, BNIP3, BTG2, CCNG1, CCNI, COPS5, CTSG, DUSP1 |
|  |  | Necrosis | 2.43E-02 | -1.031 | 45 |  |
|  |  | Cell death | 4.83E-02 | -0.947 | 61 |  |
| **Organismal, organ and tissue development** | 3:1 | Lack of mesoderm | 2.32E-02 | -2.449 | 6 | ACVR1B, COPS3, NF2, NFE2L1, SRF, STAT3 |
|  |  | Mass of hindlimb muscle | 2.43E-02 | 1.172 | 5 | HIF1AN, IFRD1, SRF, STAT5B, TRIM63, GAA, IRS1 |
|  |  | Mass of skeletal muscle | 4.54E-02 | 1.481 | 6 |  |
|  |  | Mass of muscle | 2.43E-02 | 1.784 | 7 |  |
| **Nutrient metabolism** |  | Synthesis of L-aspartic acid | 4.83E-02 |  | 2 | GOT1, GOT2 |
| **Small molecule biochemistry** |  | Metabolism of oxalacetic acid | 4.03E-02 |  | 3 | GOT1, GOT2, STAT5B |
| **L13DΔC** |  |  |  |  |  |  |
| **Cell maintenance, proliferation differentiation and replacement** | 1:0 | Proliferation of muscle cells | 1.62E-02 | 1.093 | 10 | ANG, BDNF, FGF6, mir-1, mir-15, mir-214, mir-27, NOV, SERPINB5, TFPI2 |
| **Organismal, organ and tissue development** |  | Formation of cranium | 4.43E-02 |  | 3 | COL2A1,EYA1,RUNX2 |

*between positive and negative Z-score

**at maximum 10 genes are shown
