# Supplementary material for: Immediate and long-term transcriptional response of hind muscle tissue to transient variation of incubation temperature in broilers
Source: BMC Genomics. 2016 May 4;17:323. doi: 10.1186/s12864-016-2671-9 (PMC4855815; doi:10.1186/s12864-016-2671-9)
Supplement: Additional file 4: — Assignment of DEGs to major categories, and biological functions obtained at D35 for early treatment; H10UΔC, H10DΔC, L10UΔC and L10DΔC (DOCX 22 kb) [file 12864_2016_2671_MOESM4_ESM.docx]

**Additional file 4:** Assignment of DEGs to major categories, and biological functions obtained at D35 for early treatment; H10UΔC, H10DΔC, L10UΔC and L10DΔC

| **Major category** | **Ratio*** | **Biological function** | **BH P-value** | **Z-score** | **Total DEGs** | **DEGs assigned to biofunction**** |
| --- | --- | --- | --- | --- | --- | --- |
| **H10UΔC** |  |  |  |  |  |  |
| **Cell maintenance, proliferation differentiation and replacement** |  | Fragmentation of microtubules | 6.01E-03 |  | 1 | TUBB, NCKAP1, mir-451, DPAGT1, NDFIP2, NCKAP1 |
|  |  | Delay in initiation of migration of endodermal cells | 6.01E-03 |  | 1 |  |
|  |  | Maturation of erythroblasts | 1.01E-02 |  | 1 |  |
|  |  | Assembly of intercellular junctions | 3.24E-02 |  | 2 |  |
|  |  | Nucleation of actin filaments | 3.46E-02 |  | 1 |  |
| **Organismal, organ and tissue development** |  | Development of axial skeleton | 2.37E-03 |  | 3 | HES7, TRAF4, PITX1, NCKAP1 |
|  |  | Formation of rib | 4.03E-03 |  | 2 |  |
|  |  | Growth of hindlimb | 6.01E-03 |  | 1 |  |
|  |  | Formation of vertebrae | 6.58E-03 |  | 2 |  |
|  |  | Somitogenesis | 7.11E-03 |  | 2 |  |
| **Nutrient metabolism** |  | Biosynthesis of dolichol | 6.01E-03 |  | 1 | DPAGT1 |
|  |  | Synthesis of oligosaccharide-diphosphodolichol | 1.73E-02 |  | 1 |  |
|  |  | Metabolism of UDP-N-acetylglucosamine | 2.11E-02 |  | 1 |  |
| **H10DΔC** |  |  |  |  |  |  |
| **No data above Threshold** | | |  |  |  |  |
| **L10UΔC** |  |  |  |  |  |  |
| **Cell maintenance, proliferation differentiation and replacement** | 2:0 | Colony formation | 2.97E-02 | 0.152 | 6 | ATIC,EPCAM,ERBB3,FGFBP1,mir-25, AHNAK,AHSG,AVPR2,BDKRB2,CHRM3 |
|  |  | Proliferation of cells | 4.79E-02 | 0.641 | 24 |  |
| **Organismal, organ and tissue development** |  | Contractility of ileal smooth muscle | 4.26E-03 |  | 2 | NOG,HOXA1,PITX1,TFAP2A,AVPR2,BDKRB2,CHRM2,CHRM3,CLDN2 |
|  |  | Formation of skull | 8.50E-03 |  | 4 |  |
|  |  | Osmolality of urine | 1.95E-02 |  | 3 |  |
|  |  | Size of embryonic tissue | 1.95E-02 |  | 3 |  |
|  |  | Formation of craniofacial skeleton | 2.20E-02 |  | 2 |  |
| **Nutrient metabolism** |  | Synthesis of polyols | 2.10E-02 |  | 4 | BDKRB2,CHRM2,FABP1,CHRM3,DPAGT1,GNG2,KIAA1598,NADH |
|  |  | Biosynthesis of dolichol | 2.97E-02 |  | 1 |  |
|  |  | Export of lysophosphatidic acid | 2.97E-02 |  | 1 |  |
|  |  | Hydrolysis of phosphatidylinositol | 3.93E-02 |  | 3 |  |
|  |  | Accumulation of phosphatidic acid | 3.93E-02 |  | 2 |  |
| **Genetic information and nucleic acid processing** |  | Binding of AP2 binding site | 3.93E-02 |  | 1 | TFAP2A, BDKRB2 |
|  |  | Mutation of mitochondrial DNA | 4.79E-02 |  | 1 |  |
| **Cell signaling and interaction** | 1:0 | Accumulation of cyclic AMP | 3.60E-02 | 0.686 | 4 | AVPR2, CHRM2, CHRM3, IRF6 |
| **Small molecule biochemistry** |  | Oxidation of xanthine | 3.93E-02 |  | 1 | NADH |
| **Response to stimuli** |  | Acute phase reaction | 2.97E-02 |  | 2 | MBL2, AHSG, BDKRB2, HPGDS |
|  |  | Inflammation of limb | 3.70E-02 |  | 2 |  |
|  |  | Neutralization of Influenza A virus | 4.79E-02 |  | 1 |  |
| **L10DΔC** |  |  |  |  |  |  |
| **Genetic information and nucleic acid processing** | 0:5 | Transcription | 1.57E-02 | -2.593 | 88 | MYOD1,RNPS1,SOX6,KHDRBS1,PPARGC1A,AEBP2,ARNTL,BHLHE40,BRD3,CASK |
|  |  | Transcription of RNA | 1.57E-02 | -2.303 | 86 |  |
|  |  | Transcription of DNA | 4.50E-02 | -1.095 | 15 |  |
|  |  | Splicing of mrna | 2.54E-02 | -0.692 | 10 |  |
|  |  | Processing of mrna | 1.57E-02 | -0.61 | 68 |  |

*between positive and negative Z-score

**at maximum 10 genes are shown
