# Supplementary material for: Immediate and long-term transcriptional response of hind muscle tissue to transient variation of incubation temperature in broilers
Source: BMC Genomics. 2016 May 4;17:323. doi: 10.1186/s12864-016-2671-9 (PMC4855815; doi:10.1186/s12864-016-2671-9)
Supplement: Additional file 5: — Assignment of DEGs to major categories, and biological functions obtained at D35 for late treatment; H13UΔC, H13DΔC, L13UΔC and L13DΔC. (DOCX 23 kb) [file 12864_2016_2671_MOESM5_ESM.docx]

**Additional file 5:** Assignment of DEGs to major categories, and biological functions obtained at D35 for late treatment; H13UΔC, H13DΔC, L13UΔC and L13DΔC.

| **Major category** | **Ratio*** | **Biological function** | **BH P-value** | **Z-score** | **Total DEGs** | **DEGs assigned to biofunction**** |
| --- | --- | --- | --- | --- | --- | --- |
| **H13UΔC** |  |  |  |  |  |  |
| **No data above threshold** |  |  |  |  |  |  |
| **H13DΔC** |  |  |  |  |  |  |
| **Cell maintenance, proliferation differentiation and replacement** | 1:1 | Formation of filopodia | 3.25E-02 | -0.849 | 5 | FGD3, MTBP, PKP1, RHOF, SDC3 |
|  |  | Apoptosis | 3.25E-02 | 1.253 | 29 | ADRA1A, ATF4, B4GALT1, BTG2, CX3CL1, DHODH, FABP1, FOXA2, GMCL1, GNG2 |
| **Organismal, organ and tissue development** | 2:5 | Development of cardiovascular system | 4.76E-02 | -1.546 | 15 | B4GALT1, CX3CL1, ID1, FOXA2, RBP4, SALL1, TBX3, ADRA1A, AHSG, ATF4 |
|  |  | Quantity of monocytes | 2.37E-02 | -1.432 | 5 |  |
|  |  | Concentration of hormone | 4.40E-02 | -1 | 9 |  |
|  |  | Development of body trunk | 8.37E-03 | -0.902 | 19 |  |
|  |  | Development of genitourinary system | 3.25E-02 | -0.832 | 15 |  |
|  |  | Quantity of phagocytes | 4.01E-02 | 0.131 | 9 | CX3CL1, AHSG, APOA4, ATF4, B4GALT1, FABP1, FFAR2, FOXA2, GATM, GNMT |
|  |  | Glucose tolerance | 7.74E-03 | 0.396 | 9 |  |
| **Nutrient metabolism** | 0:5 | Metabolism of carbohydrate | 7.21E-04 | -2.791 | 17 | APOA4, FOXA2, NQO1, PTGER4, SLC27A1, ADRA1A, B4GALT1, CX3CL1, GATM, GNMT |
|  |  | Synthesis of carbohydrate | 1.70E-02 | -2.789 | 11 |  |
|  |  | Quantity of carbohydrate | 1.87E-02 | -2.453 | 12 |  |
|  |  | Concentration of lipid | 1.86E-02 | -1.806 | 16 |  |
|  |  | Concentration of acylglycerol | 2.71E-02 | -1.532 | 9 |  |
| **Genetic information and nucleic acid processing** |  | Homotetramerization of protein | 1.70E-02 |  | 4 | GNMT, PCBD1, PPAT, SHMT1 |
| **Molecular transport** |  | Secretion of lactic acid | 2.71E-02 |  | 2 | CX3CL1, SLC27A1, NQO1, PTGER4, SGMS2, SNCG, SYK |
|  |  | Quantity of NADH | 3.06E-02 |  | 2 |  |
|  |  | Secretion of protein | 4.35E-02 |  | 5 |  |
| **Small molecule biochemistry** | 0:1 | Oxidation of lipid | 3.25E-02 | -1.628 | 7 | ACAA2, FABP1, IL15, NADH, NR4A3, SLC27A1, SNCG |
| **L13UΔC** |  |  |  |  |  |  |
| **Cell maintenance, proliferation differentiation and replacement** | 5:5 | Apoptosis of muscle cells | 7.19E-03 | -1.294 | 8 | CAV3, MYC, PIM1, APOA1, CASP1, IGF1R, S100A6, TFPI2, TNNT2, CEBPB |
|  |  | Cell death of muscle cells | 8.04E-03 | -1.081 | 9 |  |
|  |  | Transmembrane potential | 2.18E-02 | -0.908 | 6 |  |
|  |  | Necrosis | 2.72E-03 | -0.685 | 26 |  |
|  |  | Size of muscle cells | 1.04E-02 | -0.447 | 5 |  |
|  |  | Proliferation of fibroblast cell lines | 3.00E-02 | 2.018 | 8 | IGF1R, MYC, CEBPB, TGM2, CLU, MMP9, NFKB2, RHOB, CASP1, PIM1 |
|  |  | Cell death of connective tissue cells | 1.69E-02 | 2.139 | 10 |  |
|  |  | Cell viability | 3.00E-02 | 2.162 | 11 |  |
|  |  | Migration of cells | 4.19E-02 | 2.422 | 18 |  |
|  |  | Cell survival | 3.00E-02 | 2.68 | 13 |  |
| **Organismal, organ and tissue development** | 5:2 | Quantity of macrophages | 3.00E-02 | -1.71 | 5 | MMP9, TGM2, APOA1, CASP1, CEBPB, FABP1, IGF1R, INHA, mir-221, MYC |
|  |  | Survival of organism | 3.00E-02 | -0.09 | 11 |  |
|  |  | Quantity of cells | 8.25E-03 | 1.683 | 21 | MMP9, CLU, CEBPB, IGF1R, TNNT2, APOA1, CAV3, DES, EMX2, INHA |
|  |  | Development of body trunk | 1.92E-02 | 1.744 | 15 |  |
|  |  | Contractility of cardiac muscle | 1.81E-02 | 1.98 | 5 |  |
|  |  | Proliferation of mammary epithelial cells | 2.24E-03 | 1.982 | 4 |  |
|  |  | Contractility of heart | 3.53E-03 | 2.213 | 7 |  |
| **Nutrient metabolism** | 5:3 | Concentration of lipid | 7.66E-03 | -1.005 | 15 | APOD, CASP1, CAV3, CEBPB, FABP1, MYC, APOA1, FABP3, MMP9, RGS4 |
|  |  | Concentration of triacylglycerol | 3.00E-02 | -0.404 | 7 |  |
|  |  | Concentration of fatty acid | 7.25E-04 | -0.38 | 10 |  |
|  |  | Release of lipid | 9.51E-03 | 0.236 | 7 | APOA1, CEBPB, IGF1R, MYC, NPY, APOD, FABP3, GPC1, GFPT1, CAV3 |
|  |  | Metabolism of carbohydrate | 3.05E-02 | 0.299 | 10 |  |
|  |  | Uptake of lipid | 3.00E-02 | 0.9 | 5 |  |
|  |  | Uptake of monosaccharide | 8.81E-03 | 1.067 | 8 |  |
|  |  | Uptake of D-glucose | 1.06E-02 | 1.706 | 7 |  |
| **Genetic information and nucleic acid processing** | 1:0 | Synthesis of DNA | 1.12E-02 | 0.088 | 9 | CLU, IGF1R, INHA, MYC, PIK3IP1, PIM1, RGS4, SUPT4H1, TFPI2 |
| **Molecular transport** |  | Transmembrane transport of ion | 3.00E-02 |  | 3 | CHRNG, MYC, P2RX5, SAT1, S100A6 |
|  |  | Entrance of DNA | 3.00E-02 |  | 1 |  |
|  |  | Depletion of spermine | 3.00E-02 |  | 1 |  |
| **Cell signaling and interaction** | 1:0 | Protein kinase cascade | 3.39E-02 | 1 | 7 | CASP1, CAV3, IGF1R, IRAK1BP1, MYC, NDFIP2, TGM2 |
| **Small molecule biochemistry** | 2:0 | Oxidation of palmitic acid | 6.62E-03 | 1.98 | 4 | APOA1, FABP3, IGF1R, SAT1, FABP1 |
|  |  | Oxidation of long chain fatty acid | 2.25E-03 | 2.213 | 5 |  |
| **Response to stimuli** | 0:3 | Inflammation of body region | 7.25E-04 | -3.154 | 19 | APOA1, CASP1, CAV3, CHRNG, CISH, CLU, IGF1R, INHA, mir-19, mir-221 |
|  |  | Inflammation of organ | 1.83E-03 | -3.03 | 20 |  |
|  |  | Inflammation of body cavity | 1.24E-02 | -2.429 | 21 |  |
| **L13DΔC** |  |  |  |  |  |  |
| **Organismal, organ and tissue development** | 0:1 | Size of body | 5.16E-03 | -4.257 | 23 | AKAP6, CERS3, CNTNAP2, DNAJA1, EYA4, GNPAT, GSTZ1, HOXD4, KHDRBS1, LAMA4 |

*between positive and negative Z-score

**at maximum 10 genes are shown
