# Supplementary material for: Immediate and long-term transcriptional response of hind muscle tissue to transient variation of incubation temperature in broilers
Source: BMC Genomics. 2016 May 4;17:323. doi: 10.1186/s12864-016-2671-9 (PMC4855815; doi:10.1186/s12864-016-2671-9)
Supplement: Additional file 6: — Body weight, carcass weight and weight of hind muscles of broilers of the experimental groups used for expression analyses. (DOCX 19 kb) [file 12864_2016_2671_MOESM6_ESM.docx]

**Additional file 6:** Body weight, carcass weight and weight of hind muscles of broilers of the experimental groups used for expression analyses.
