# Supplementary material for: Immediate and long-term transcriptional response of hind muscle tissue to transient variation of incubation temperature in broilers
Source: BMC Genomics. 2016 May 4;17:323. doi: 10.1186/s12864-016-2671-9 (PMC4855815; doi:10.1186/s12864-016-2671-9)
Supplement: Additional file 7: — Primers used for quantitative real-time PCR (qPCR) (DOCX 15 kb) [file 12864_2016_2671_MOESM7_ESM.docx]

| **Additional file 7**  Primers used for quantitative real-time PCR (qPCR) | | | | | | | |  |  | |  | | |  | | |  |
| --- | --- | --- | --- | --- | --- | --- | --- | --- | --- | --- | --- | --- | --- | --- | --- | --- | --- |
|  |  | |  | |  | |  | | |  | | |  | |  | | |
| Gene Symbol | Gene  name | Accession  no. | | Primer F | | | Primer R | | | | |  | | | |  |  |
| GAPDH* | glyceraldehyde-3-phosphate dehydrogenase | NM_204305 | | AGTCGGAGTCAACGGATTTG | | | CTGCCCATTTGATGTTGCTG | | | | |  | | | |  |  |
| ACTB* | actin, Beta | NM_205518 | | CCTCTTCCAGCCATCTTTCTT | | | TAGAGCCTCCAATCCAGACA | | | | |  | | | |  |  |
| NR4A3 | nuclear receptor subfamily 4, group A, member 3 | ENSGALT  00000022087 | | GACAGCCTGAAAGGGAGAAG | | | CGGTGGAACAGTATCGTGAATA | | | | |  | | | |  |  |
| FGA | fibrinogen alpha chain | ENSGALT  00000015077 | | CAAGCCAGAGGGATCCAATAAG | | | ACAACTCTCCTTGCCCTTTG | | | | |  | | | |  |  |
| AHSG | alpha-2-HS-glycoprotein | ENSGALT  00000014007 | | CATGGATACAGGTTTGCCTTAAAC | | | TAACATCACAGTCACCCTCAAC | | | | |  | | | |  |  |
| * Housekeeping gene | | |  | |  |  | | |  | |  | | |  | | |  |
|  |  | |  | |  |  | | |  | |  | | |  | | |  |
